# An Intervention to Increase Condom Use Among Users of Sexually Transmitted Infection Self-sampling Websites (Wrapped): Protocol for a Randomized Controlled Feasibility Trial

**DOI:** 10.2196/43645

**Published:** 2023-05-11

**Authors:** Katie Newby, Kayleigh Kwah, Lauren Schumacher, Rik Crutzen, Julia V Bailey, Louise J Jackson, Stephen Bremner, Katherine E Brown

**Affiliations:** 1 Department of Psychology, Sport and Geography School of Life and Medical Sciences University of Hertfordshire Hatfield United Kingdom; 2 Department of Health Promotion Maastricht University Maastricht Netherlands; 3 eHealth Unit Department of Primary Care and Population Health University College London London United Kingdom; 4 Institute of Applied Health Research College of Medical and Dental Sciences University of Birmingham Birmingham United Kingdom; 5 Department of Primary Care and Public Health Brighton and Sussex Medical School Brighton United Kingdom

**Keywords:** adolescent, condom use, digital health, eHealth, feasibility randomized controlled trial, sexual health, sexually transmitted infections, STIs, self-testing, young people

## Abstract

**Background:**

Reducing the rates of sexually transmitted infections (STIs) among young people is a public health priority. The best way to avoid STIs from penetrative sex is to use a condom, but young people report inconsistent use. A missed opportunity to intervene to increase condom use is when young people access self-sampling kits for STIs through the internet. The potential of this opportunity is enhanced by the increasing numbers of young people being tested through this route every year in England. Hence, in a cocreation by young people, stakeholders, and researchers, Wrapped was developed––a fully automated, multicomponent, and interactive digital behavior change intervention developed for users of STI self-sampling websites, who are aged 16-24 years.

**Objective:**

This paper is a protocol for a feasibility randomized controlled trial (fRCT). The fRCT seeks to establish whether it is feasible to run a randomized controlled trial to test the effectiveness and cost-effectiveness of Wrapped. Wrapped aims to reduce the incidence of STIs through increasing correct and consistent use of condoms among users of STI self-sampling websites, who are aged 16-24 years.

**Methods:**

A 2-arm parallel-group randomized fRCT of Wrapped plus usual care, compared to usual care only (basic information on STIs and condom use), with a nested qualitative study. A minimum of 230 participants (aged 16-24 years) are recruited from an existing chlamydia self-sampling website. Participants are randomized into 1 of 2 parallel groups (1:1 allocation). Primary outcomes are the percentage of users recruited to the fRCT and the percentage of randomized participants who return a chlamydia self-sampling kit at month 12. Additionally, besides chlamydia positivity based on biological samples, surveys at baseline, month 3, month 6, and month 12, are used to assess condom use attitude, behavioral capability, self-efficacy, and intention, along with details of any partnered sexual activity and condom use, and health economic data. Nested qualitative interviews with trial participants are used to gain insight into the factors affecting recruitment and attrition.

**Results:**

Recruitment to the fRCT began in March 2021 and was completed in October 2021. Data collection was completed in December 2022.

**Conclusions:**

This feasibility study will provide data to inform the design of a future-definitive trial. This work is timely given a rapid rise in the use of internet testing for STIs and the sustained high levels of STIs among young people.

**Trial Registration:**

ISRCTN Registry ISRCTN17478654; http://www.isrctn.com/ISRCTN17478654

**International Registered Report Identifier (IRRID):**

DERR1-10.2196/43645

## Introduction

### Background and Rationale

Sexually transmitted infection (STI) diagnoses in England have been persistently high over the past decade [1]. Although the most recent annual data show a 32% reduction in STI diagnoses, down from a 10-year high of 467,096 in 2019 to 307,901 in 2020, this likely reflects a temporary shift brought about by the COVID-19 pandemic, which placed restrictions on public behavior and reduced access to STI testing [1,2]. Young people continue to carry a high burden of infection, with 46% of all new diagnoses among 15- to 24-year-olds [1]. Furthermore, women, those of Black ethnicity, those living in the most deprived areas, and men who have sex with men, remain disproportionately affected [1]. Among young people, chlamydia continues to be the most prevalent STI [1]. Rates of chlamydia among 15- to 24-year-olds have been high but stable over recent years, whereas increases have been observed for other STIs, such as gonorrhea and syphilis [2]. The removal of COVID-19 restrictions and an increase in testing availability will likely bring with it a return to prepandemic levels of STI diagnoses and as such, sexual health continues to be a public health concern [1]. Untreated STIs can lead to serious health consequences, such as pelvic inflammatory disease, ectopic pregnancy, and infertility, which have a significant impact on health and quality of life [3]. The estimated annual cost to the National Health Service (NHS) of STI treatment is £620 (US $762.6) million [4]. The best way to avoid STIs from penetrative sex is to use a condom [5], but young people report inconsistent use [6,7].

Although condom use is the cornerstone of STI prevention, STI testing is important for treatment and to prevent onward transmission. There have been notable changes in the landscape of STI testing within England since 2017. In the period between 2017 and 2019, against a backdrop of increasing testing overall, there was a shift toward the use of internet services, and away from other nonspecialist services [8]. Between 2018 and 2019, while the number of tests carried out by specialist sexual health services (eg, genitourinary medicine services) remained relatively stable, those carried out by nonspecialist services (excluding internet testing), such as pharmacies, general practice, and community-based settings, decreased by 9%, and those by internet services increased by 69% [8]. This trend reflects recent year-on-year increases, with internet testing accounting for 4% of all testing in 2017, 13% in 2018, and 19% in 2019 [8]. The global COVID-19 pandemic has further served to accelerate the move in this direction across Europe [9,10]. In England, despite there being a reduction of 30% in expected levels of chlamydia testing in 2020, a 2-fold increase in internet testing was observed, with approximately 40% of all chlamydia testing being processed through the internet in 2020 [1]. Whether internet testing remains at these elevated levels or begins to adjust back toward those observed prepandemic, remains to be seen. Regardless, internet testing is now an established and significant route to STI diagnosis and treatment within England.

Evidence indicates that STI self-sampling websites are effective at reaching higher prevalence groups; chlamydia positivity is comparable to that of other nonspecialist services [8], and they attract equivalent numbers of males and females, and those from across the spectrum of deprivation (E Hollis, unpublished data, 2017). Repeated use of internet testing services is however common, with chlamydia positivity remaining high for those that retest (E Hollis, unpublished data, 2017), suggesting that testing online does not reliably translate into future individual prevention efforts. Furthermore, while information on STIs and contraceptive methods is typically found on self-sampling websites, this is insufficient for behavior change [11], and inconsistent with Public Health England guidance to provide those at elevated risk of STIs with preventative intervention [12]. As such, self-sampling websites provide an important but currently missed opportunity to intervene with a priority group.

In 2016-2017, with the support of Medical Research Council Public Health Intervention Development funding and in line with Medical Research Council guidance on the development of complex interventions [13], Wrapped was cocreated by young people, stakeholders, and researchers [14]. Wrapped is a fully automated, multicomponent, and interactive digital intervention developed for the users of STI self-sampling websites aged 16-24 years. It aims to reduce the incidence of future STIs by increasing correct and consistent use of condoms for penetrative sex. The users of STI self-sampling websites can be signposted to Wrapped upon order of an STI self-sampling kit (through a hyperlink). Content is tailored, with between 1 and 6 different components allocated to users in accordance with their self-identified barriers to condom use. These barriers reflect important behavioral determinants of condom use identified through existing meta-analyses [15-18], namely condom use attitudes (particularly beliefs around pleasure, enjoyment, and spontaneity), condom availability, and behavioral capability and self-efficacy for condom use and communication.

In 2019, funding was awarded by the National Institute for Health and Care Research Public Health Research program to determine whether it is feasible to run a randomized controlled trial (RCT) to test the effectiveness and cost-effectiveness of Wrapped. This paper presents the protocol for this feasibility randomized controlled trial (fRCT).

### Aim and Objectives

The aim of this study is to assess whether and how it is possible to carry out a future definitive RCT to evaluate the effectiveness and cost-effectiveness of Wrapped plus usual care, in comparison to usual care only.

The primary objectives are to estimate the following parameters for planning a definitive RCT:

1. The rate of recruitment of eligible participants.
2. The rate of participant follow-up for the definitive RCT primary outcome measure (chlamydia positivity at 12 months measured using biological samples).

The secondary objectives are as follows:

1. Estimate the rate of participant follow-up for the definitive RCT secondary outcome measure chlamydia cumulative incidence.
2. Identify whether the level of deprivation of the final sample is representative of web-based STI self-sampling users.
3. Identify whether differential retention occurs across groups (gender, ethnicity, sexual identity, deprivation, randomized groups, and chlamydia diagnosis at baseline).
4. Measure chlamydia positivity at 12 months in the intervention and control groups (to support sample size calculation for the definitive RCT).
5. Identify the rate of attrition at 3 months, 6 months, and 12 months, and ways of minimizing this.
6. Determine the feasibility and participant acceptability of all primary and secondary outcome measures (including health economic measures).
7. Identify which recruitment messages result in the highest rate of recruitment.
8. Identify and remove the intervention “friction points” (using web analytics data) to minimize attrition and maximize the future intervention dose.
9. Identify costs and resource use associated with the intervention for health care services and users (to inform the design of the definitive RCT and the future economic evaluation).
10. Measure contamination of intervention effect in the control group.
11. Identify possible adverse effects of the intervention, for example, participants’ inadvertent disclosure that they are testing for an STI or increase in consumption of pornography.

### Study Design

A 2-arm parallel group fRCT of Wrapped plus usual care compared to usual care alone with a nested qualitative study.

## Methods

Please refer to Multimedia Appendix 1 for a visual representation of the study design, recruitment process, and timing of measures based on the Consolidated Standards of Reporting Trials (CONSORT) extension for pilot and feasibility trials [19].

### Study Population and Setting

Recruitment, intervention delivery, and data collection are conducted entirely on the internet. Participants will be recruited from an existing chlamydia self-sampling website (freetest.me [20]). Freetest.me provides a web-based chlamydia testing service to local authority areas across England (typically subcontracted to and administered through NHS trusts) and is free at the point of use. For the purposes of this study, users of freetest.me residing in 1 of 4 local authority areas will be invited to participate.

### Eligibility Criteria

The inclusion and exclusion criteria are outlined in Textbox 1.

**Inclusion and exclusion criteria.**

**Inclusion criteria:**

- Aged 16-24 years.
- Living in a local authority area participating in the study.

**Exclusion criteria:**

- No internet access.
- Sexual activity does not typically include penetrative sex (penis in vagina or anus).

### Participant Timeline

Table 1 gives an overview of participant activities and timing.

To recompense for time spent in completing activities, participants will receive payment in the form of vouchers (see Multimedia Appendix 2 for the schedule of payments).

**Table 1:** Participant activities across the duration of the study.

Activity	M0^a^	M3^b^	M6^c^	M12^d^
Read participant information	✓			
Complete consent	✓			
Complete survey	✓	✓	✓	✓
Self-report chlamydia test result	✓			
Complete chlamydia self-sample		✓		✓

^a^M0: month 0.

^b^M3: month 3.

^c^M6: month 6.

^d^M12: month 12.

### Recruitment

A study invitation will be placed on the “thank you” page of the freetest.me website displayed to users after they have placed an order for a chlamydia self-sampling kit. Every 2 weeks, the advert will change to emphasize a different value proposition (see Multimedia Appendix 3 for the list of adverts). A hyperlink within the advert will take users to Research Electronic Data Capture (REDCap), a secure data capture and management platform, for participant information and consent (see Multimedia Appendix 4 for a copy of the participant information and consent form). Each individual’s freetest.me order number will be appended to the URL. This order number will be captured by REDCap enabling the research team to ensure that each participant is a unique and genuine user of freetest.me.

### Survey Completion (Months 0, 3, 6, and 12)

REDCap is being used to monitor and manage all survey data collected as part of this study. Surveys will be conducted at baseline (month 0 [M0]), month 3 (M3), month 6 (M6), and month 12 (M12) and used to collect data on condom use attitude, behavioral capability, self-efficacy, and intention, along with details of any partnered sexual activity and condom use that has occurred in the previous 3-month period. The surveys will also be used to measure the experience of partner resistance to condom use and to collect health economic data. There are between 63 and 76 core items per survey (depending on time point), with data largely being collected using fixed response items and Likert scales (see Multimedia Appendix 5 for survey items). The baseline survey will additionally collect participant’s contact details to enable communication with participants regarding the completion of follow-up surveys, and basic demographic data. The M3 survey will also be used to collect self-reported data on participant engagement with the intervention.

Completion of the baseline survey is scheduled immediately after consent is provided. All other survey completion will be triggered by email. Participants who do not complete the survey will be sent up to 3 reminders (delivered by SMS text message). Subsequently, if no response is received, 1 email and 1 telephone call will be made to prompt completion. During the telephone call, participants will be given the option to verbally respond to a reduced set of survey questions. Finally, if there is still no response, a paper-based version of the survey will be posted out with a stamped addressed return envelope. Those who have not provided any data 20 days after the initial survey is sent out will be classed as nonresponder. Surveys will continue to be sent to nonresponders (as well as chlamydia tests––see below) at subsequent time points unless a participant asks to be withdrawn from the study.

### Chlamydia Testing (M0, M3, and M12)

Chlamydia tests will be processed by freetest.me at M0, M3, and M12. The chlamydia test at M0 is triggered by participants when they order a self-sampling kit through the freetest.me website immediately before receiving the study invite (see “recruitment” above). Participants will be asked to self-report their result, and any treatment received if applicable, through SMS text message. A single SMS text message reminder will be sent to participants who do not respond. Those who have not provided this data 4 weeks after the SMS text message contact is made will be classed as nonresponders.

The chlamydia tests at M3 and M12 will be processed differently to that at M0. For this purpose, chlamydia self-sampling kits will be sent directly from the study team to participants. One week before posting the kits, participants will be sent an SMS text message asking them to confirm or amend their postal address. The kits that participants receive at M3 and M12 will contain the usual user instructions to collect the sample and return by Freepost to freetest.me for processing. Participants will be sent 3 SMS text message reminders to complete the test kit. If the test kit still has not been returned after this point, 1 phone call will be made to prompt return. Those who have not returned their kit 30 days after it is posted out will be classed as a nonresponder. Participants who have a positive test result at M0, M3, or M12 will receive 1 item within SMS text message survey 2 weeks post results to record whether their infection has been treated or not. If treatment is incomplete, or the participant does not respond, they will be sent 1 reminder SMS text message to obtain this information. Each local NHS trust will be informed of any positive cases (for participants residing in their local authority area) so that they can provide appropriate treatment and follow-up.

### Randomization

Randomization of participants to trial groups in a ratio of 1:1, is fully automated, and fully concealed within REDCap software, and will take place following completion of the M0 survey. Before randomization of each participant, the research team will manually check that each individual is unique, by cross-checking their identifying information (name, email address, postal address, mobile phone number, and freetest.me order number) with participants already within the sample. They will also check that the participant has answered the baseline survey with reasonable care (ie, no evidence of random responding or completion under the expected minimum time). Those not meeting these criteria will not be randomized. Participants will be blind to group allocation and the research team will be unable to influence any aspect of this procedure. The randomization list will be generated by the study statistician who will monitor randomization on a weekly basis during active recruitment to ensure that the algorithm is being correctly applied. Randomization will be at the individual level as control group contamination is not anticipated (although this will be monitored to determine the suitability of this approach for the definitive trial). Stratification across groups, using randomly permuted blocks (ethnicity, sexual identity, deprivation) will be performed to balance participants across the trial arms.

### Intervention Access, Content, and Tracking

Participants will be individually randomized within the stratifications into 1 of 2 groups: usual care or Wrapped plus usual care (see “Wrapped website” and “comparator website” below for further details on the respective interventions). Participants will be directed to their allocated intervention following randomization through a hyperlink. All participants will be free to interact as much or as little with the respective materials; participants will be emailed the hyperlink to enable repeat visits. An email reminder to visit the intervention will be sent to all participants who have not done so 2 weeks after first access is provided.

#### Wrapped Website

See Multimedia Appendix 6 for the intervention logic model which presents the theoretical model and an outline of intervention components. Newby et al [14] have provided a detailed description of intervention content reported in line with Template for Intervention Description and Replication guidance and have provided screenshots, images of intervention materials, and example videos.

#### Comparator Website

Usual care is the comparator. Existing STI self-sampling websites typically provide only basic information on STIs and condom use to their users [21]. To replicate this level of health promotion, and to provide an equivalent “intervention” experience for those in the control group, a stand-alone WordPress website presenting comparable basic information has been created [22]. The same basic information is also provided on the Wrapped website.

#### Analytics Data

Participant access to and engagement with their allocated website will be tracked. There is no minimum acceptable level of engagement––all participants will be allowed to continue in the trial regardless of their level of interaction with the material. Tracking will occur in the background (with each participant’s explicit consent) to ensure that it is unobtrusive. Data will be collected within the Wrapped website itself using an analytics package called Matomo, a secure open-source platform [23] (see Multimedia Appendix 7 for details on what will be measured). Analytics data will be used to calculate intervention dose received and to identify any fixable “friction points” in the user journey that may adversely affect usability.

### Primary Outcome Measures

Primary outcome measures are as follows:

1. The percentage of freetest.me users recruited to the feasibility RCT.
  This will be assessed as the number of participants randomized out of those using freetest.me in the 4 local authority areas, that is, all those exposed to the recruitment advert.
2. The percentage of randomized participants with a valid primary outcome measure. This will be assessed as the percentage of randomized participants who provide a chlamydia test result at M12.

### Secondary Outcome Measures

Secondary outcome measures are as follows:

1. Percentage of randomized participants with outcome data required to measure cumulative incidence of chlamydia (measured using M3 and M12 chlamydia self-sample, and the relevant item within M3, M6 and M12 surveys).
2. The distribution of Index of Multiple Deprivation quintile among users of freetest.me over the 3-month recruitment period, compared to that of the final sample, within each of the 4 recruitment areas.
3. The number of randomized participants with a valid primary outcome measure at M12 by group (gender, ethnicity, sexual identity, deprivation, randomized groups, and chlamydia diagnosis at baseline).
4. The number of randomized participants testing positive for chlamydia at M12 in the intervention and the control group.
5. Attrition curves comparing the percentage of valid participants in the trial at randomization, M3, M6, and M12 plotted for the intervention and control arms (dropout attrition).
6. Completeness of data from outcome measures that would be needed in a definitive trial (ie, self-report of chlamydia result at baseline, results from biological samples, demographic information, self-report of condom use and chlamydia infection, and data needed for cost-effectiveness and cost-utility analyses).
7. The percentage of freetest.me users randomized following each of the different recruitment messages employed.
8. The percentage of valid participants in the 2 arms that do not achieve predetermined intervention “goals” (ie, do not follow instructions within each component of the Wrapped website to their culmination, for example, ordering a sample pack, or watching a suggested video), and the bounce rate for home and content pages.
9. Completeness of data on costs and resource use that would be needed for the economic evaluation in the definitive RCT.
10. Proportion of participants in the control group who report (at M12) any exposure to Wrapped.
11. The total number of adverse events reported during the course of the study (inadvertent disclosure that sexually active or testing for STI if overseen using Wrapped, initiation or change in the consumption of pornography, or other).

### Sample Size

At least 60 participants per arm are recommended to allow proportions to be estimated with good precision [24]. This is inflated to allow for an estimated 25% nonreturn of initial chlamydia self-test sample (based on freetest.me statistics; 60/0.75=80) and a further estimated 30% dropout over the course of the feasibility trial (a conservative estimate based on 19% dropout achieved in Free et al [25] sexual health feasibility RCT (80/0.7=114 per arm). Based on this, the total sample size required for the present fRCT has been calculated as 230 participants.

### Progression Criteria

The following criteria will be used to inform progression to a full trial:

1. The proportion of freetest.me users recruited to the feasibility trial is sufficient to obtain the sample size required for the definitive RCT (sample size for the definitive RCT will be determined using data from multiple sources, including chlamydia positivity in the control group of the present fRCT and national level data on chlamydia positivity among young people testing for STIs through the internet (used to estimate the level of chlamydia positivity in the target population), expert opinion on what would be a clinically meaningful reduction in chlamydia positivity for the target population, typical effect sizes achieved in comparable studies, and the size of the available sampling pool for the future RCT).
2. A total of 60% (138/230) of participants (those randomized) followed up for the definitive RCT primary outcome measure at M12
3. Index of Multiple Deprivation quintile distribution for the final sample is comparable to that of individuals in the sampling pool (freetest.me users in the partner local authority areas).
4. Adverse events are judged as sufficiently infrequent and/or serious to cause concern.

The progression decision will be determined by the independent Study Steering Committee (SSC), in conjunction with the Data Monitoring and Ethics Committee, based on achievement of all the above criteria, or convincing evidence that any 1 criterion is amenable for necessary improvement.

### Ethical Considerations

Ethical approval was obtained from the NHS East Midlands—Leicester Central Research Ethics Committee (reference 20/EM/0275) and the trial was registered with the ISRCTN (ISRCTN17478654). To participate, all individuals will be required to provide informed consent. All participants will receive a £65 (US $79.95) payment in the form of vouchers to recompense for time spent completing the research activities. All research data will be deidentified before analysis and reporting.

### Data Management and Analysis

#### Data Management

All trial data will be collected in REDCap, a secure data management program hosted on a university server. REDCap will create a unique ID for each participant at the point of consent. All activity will be associated with this unique ID, enabling data across time points to be easily and accurately linked. Analytics data will be stored within Matomo and the Wrapped website, also linked to the unique ID. Only the research team will have access to data stored on REDCap, Matomo, and the Wrapped website. At the end of data collection, all data captured through these platforms will be exported to a password-protected folder for analysis purposes. Only the research team will have access to this folder. Consent data will be separated from all other data (research data) through storage in 2 separate folders. For the research data, at the point of export, all identifying data will be removed and the remaining data linked using the unique participant ID before cleaning. Once the research team agrees that all data is present and complete, all data present in REDCap, Matomo, and the Wrapped website will be permanently deleted. Research data will remain in the password-protected folder until analysis is complete after which it will be deleted. Consent data will be kept for 6 years before deletion. In line with the funder’s requirements, a copy of the raw anonymized research data will be deposited within the University of Hertfordshire Research Archive (UHRA [26]) and made available indefinitely on an open-access basis.

#### Statistical Analysis

Researchers performing the analysis (KN, KB, LJ, and SB) will be blinded to participant allocation. Quantitative data will be analyzed descriptively. Measures of location and dispersion, including means and SDs, medians and IQRs, and number and percent for categorical variables, will be used to describe the data at baseline and at each follow-up time point. CIs will be calculated and presented as appropriate. All analyses will be performed using SPSS (version 28; IBM Corp). As with the raw data, the syntaxes used for analysis and the output generated will also be deposited within the UHRA and made available indefinitely on an open-access basis.

### Nested Qualitative Study

A nested qualitative study will be conducted during the fRCT. The aims of this study will be to explore participant’s views and experiences of the Wrapped intervention, experience of trial procedures and materials, and perceptions of whether and how the intervention has changed their condom use beliefs or behavior.

Approximately 30 individuals (number to be determined by data saturation) participating in the fRCT will be invited to take part. All participants will receive a £20 (US $24.6) voucher in recognition of their time. Participants will be asked at baseline (single question in survey) whether they are willing to take part in a semistructured telephone or video call interview and if so, to provide suitable contact details.

Participants will be purposively selected to create a varied sample representing different demographic criteria, completers and noncompleters from both trial arms, those with varying levels of intervention engagement, and those thought to have experienced specific events that could influence dropout. Selected individuals will be contacted and directed to a new project page on REDCap where they will be asked to provide informed consent to take part in this additional aspect of the study.

Interviews will last up to 1 hour and will be fully transcribed before analysis. Framework analysis [27] will be used to analyze the data.

At the end of each interview, a second aspect of the qualitative study will be discussed with the participants. This is interviewing sexual partners to better understand any changes in condom use decision-making in light of the intervention. After the interview, an email invitation will be sent to the participant which they can choose to forward to their partner if they wish. It will then be up to the sexual partner to contact the research team if they want to participate. They will be given the option to email or telephone the research team if they have any questions, or simply to follow a hyperlink straight to the relevant project page on REDCap. Here, they will be required to provide informed consent to take part in the study. All data will be collected, processed, and analyzed as described above for primary interviewees. Each individual who participates will be given a £20 (US $24.6) voucher in recognition of their time.

### Patient and Public Involvement

A patient and public involvement (PPI) group will be formed for this study. Members will be existing users of web-based STI self-sampling services. The research team will work with the PPI group to determine the different value propositions included in the study advertisements; determine strategies for effective retention of participants (eg, the level and timing of voucher payments provided, communication methods, and tone of messaging); draft the participant information and consent statements for the fRCT and nested qualitative study to ensure that they are clear and easy to understand; iteratively test the surveys before their use to maximize content validity, readability, clarity, and comprehensiveness; review fRCT findings and reflect on changes that should be made to recruitment and retention strategies before a definitive trial; and two members of the PPI group will also support the monitoring of the fRCT by serving as members of the SSC.

### Research Governance Risks, and Dissemination

#### Monitoring

An independent SSC and Data Monitoring and Ethics Committee will be convened to provide oversight and ensure adherence to standards of best practice. Institutional sponsorship is required. A clinical trial unit will provide trial management and statistical oversight.

#### Risks, Support, and Safeguarding

Participating in the fRCT has limited risk associated with it for participants. As a result of participation in the fRCT, some people may attempt to use condoms and fail, if for example, their sexual partner responds negatively to their request to use a condom or there are condom-use errors, and this may have some negative psychological impacts in the short-term. Both the Wrapped and the comparator website will contain links to sources to help and support on topics of sex and relationships, including condom use and intimate partner violence. All participants will be provided with a debrief email at the end of their participation in the trial (at M12). Surveys at all time points will include items that measure sexual well-being. Responses may indicate that a participant was or is the victim of sexual coercion or abuse. Participants aged 18 years and over who indicate this will be signposted to appropriate sources of support by email and offered, at their request, to receive support from a university safeguarding officer. Alternatively, if a participant younger than 18 years indicates coercion or abuse, the study safeguarding procedure will be initiated. Under these circumstances, the confidentiality agreement with the participant will end, and the information provided will be passed to their local NHS trust sexual health service so that they can support them in line with existing protocols. Data-sharing agreements will be in place with these trusts to enable this action to be performed. The relevant individual will not be able to refuse this referral process, but they will be informed that this is happening. All participants are informed of this safeguarding procedure within the participant information and are required to agree to this at consent. A reminder of this process is also stated alongside the relevant questions within each survey. Participants will also be asked to disclose their experience of adverse events within M3, M6, and M12 surveys. These events could be as follows: (1) inadvertent disclosure to another person that the participant is sexually active or testing for an STI as a result of them being overseen using Wrapped, (2) initiation or change in the consumption of pornography by the participant, and (3) other. If an event is disclosed, the circumstances will be discussed by the research team who will seek advice from the sponsor, ethics committee, and/or steering committee, if required before determining the most appropriate course of action.

Participation in the nested qualitative study is low risk. Participants will be asked about their experiences of condom use before and after the intervention. It is possible that some participants may find these types of questions uncomfortable. The nature of the study is clearly outlined in the participant information and participants are made aware that they can withdraw at any time, as well as choosing not to answer any question that they are uncomfortable with. Debriefing information will be emailed to all secondary participants (sexual partners of participants in the fRCT) after the interview; primary participants will receive this on completion of the fRCT.

#### Consent

Informed consent will be taken on the internet through REDCap (separately for the fRCT and the nested qualitative study). Participants must agree to all statements before being allowed to proceed. Participants who do not agree will be thanked for their interest and routed out of the project; no data will be collected (see Multimedia Appendix 4 for a copy of the consent form).

#### Confidentiality

Individuals’ decision to take part and all their data will be treated as confidential. All data will be stored securely, and access restricted to the research team. At the point of consent, a unique identifier will be assigned, and all data linked using this. At download, personal identifying information will be removed from the data set. The only data preserved after the end of the study will be consent data (kept for 6 years) and the anonymized data set, held in a research archive indefinitely.

#### Dissemination

During the study, progress will be communicated to stakeholders through a project website and linked Twitter account. At end of the study, findings will be disseminated to the public through a written and video lay summary. Findings will be communicated to the academic community through conference presentations and publications in an open-access journal.

## Results

Recruitment to the fRCT began in March 2021 and was completed in October 2021. Approximately half of all participants have reached the M12 time point (study end) to date. Data collection was completed in December 2022.

## Discussion

### Overview

This fRCT is designed to assess whether and how it is possible to carry out a future definitive RCT to evaluate the effectiveness and cost-effectiveness of an intervention to increase condom use among young people. The rapid increase in use of web-based STI self-sampling services, accelerated by the COVID-19 pandemic, means that young people are increasingly required to self-manage their own sexual health screening. This, along with persistently high rates of STIs among this age group, makes the provision of behavior change support in this web-based context imperative.

### Strengths and Limitations

This fRCT employs the proposed design and methods that would be used in a full RCT. This will enable the research team to develop, assess, and optimize all the systems and procedures required to run the full trial before time and resource commitments are allocated. Furthermore, the study has been setup to inform important aspects of this future trial, such as which advertisements results in the highest recruitment rate? Are there any friction points within the intervention content that may contribute to dropout? Could the tone, content, or frequency of communication with participants be improved? Should the progression criteria for this feasibility fRCT be satisfactorily met, the research team will refine intervention content and study procedures in line with this to further enhance participant recruitment and retention, and study efficiency.

As this is an fRCT, the study is being conducted on a much smaller scale than a future trial. Some technical aspects of the study that are manageable at this stage will likely need to be revised. In the present study, for example, the research team are reporting positive chlamydia test results (arising from M3 and M12 testing) to each relevant trust which is time-consuming and represents an avoidable data protection risk. An alternative would be for this information to be passed directly to the trusts by the self-sampling service provider. In the future-full trial, the team would seek to overcome such technical issues.

The unusual context in which this fRCT is being run should be acknowledged. The COVID-19 pandemic has affected sexual health services and sexual behavior in ways that could not have been imagined at the time this study was conceived. When recruitment to the fRCT was initiated, restrictions on public behavior imposed to control the spread of infection were still in place but were lifted approximately 3 months later. The impact of the environment at that time on STI testing and diagnoses will not be known until the release of annual STI data in Autumn 2022. Although an increase in the use of internet testing will serve to increase the size of the sampling pool drawn upon for this study, thus potentially accelerating recruitment, it should not impact upon the recruitment rate, as this is merely a reflection of the rate at which those who see the advert are converted into participants. It should, however, be acknowledged that the pandemic is likely to have both positively and negatively impacted on people’s time and/or willingness to engage in research, factors which for some will also have changed with changing restrictions and may serve to influence study recruitment and retention. It is also important to consider whether changes in sexual risk behavior during this time could lead to fewer cases of chlamydia being identified in the present study than anticipated. This concern is, however, not supported by community surveys, which indicate that a substantial proportion of people continued to engage in at-risk behavior during 2020 [28-30], and the most recent STI data which shows that among those seeking internet testing, chlamydia positivity was comparable between 2019 (23,426/284,050, 8.2%) and 2020 (33,294/381,744, 8.7%) [1].

## Appendix

Multimedia Appendix 1 Wrapped CONSORT Diagram.

Multimedia Appendix 2 Schedule of voucher payments.

Multimedia Appendix 3 Advert schedule.

Multimedia Appendix 4 Participant information and consent.

Multimedia Appendix 5 Survey items.

Multimedia Appendix 6 Intervention logic model.

Multimedia Appendix 7 Analytics data measurements.

## Abbreviations

CONSORT: Consolidated Standards of Reporting Trials
fRCT: feasibility randomized controlled trial
NHS: National Health Service
PPI: patient and public involvement
RCT: randomized controlled trial
REDCap: Research Electronic Data Capture
SSC: Study Steering Committee
STI: sexually transmitted infection
UHRA: University of Hertfordshire Research Archive

## Data Availability

A copy of the raw anonymized research data, the syntaxes used for analysis, and the output generated, will be deposited within the University of Hertfordshire Research Archive (UHRA; accessible via https://uhra.herts.ac.uk) and made available indefinitely on an open access basis.

## References

[1] (2021). Sexually Transmitted Infections and Screening for Chlamydia in England, 2020.

[2] (2022). Sexually transmitted infections (STIs): annual data tables, 2021. Public Health England.

[3] O'Connell CM, Ferone ME (2016). Chlamydia trachomatis genital infections. Microb Cell.

[4] (2013). Unprotected nation: the financial and economic impacts of restricted contraceptive and sexual health services. Society of Sexual Health Advisers.

[5] (2013). A framework for sexual health improvement in England. Department of Health.

[6] Wellings K, Nanchahal K, Macdowall W, McManus S, Erens B, Mercer CH, Johnson AM, Copas AJ, Korovessis C, Fenton KA, Field J (2001). Sexual behaviour in Britain: early heterosexual experience. Lancet.

[7] Mercer CH, Tanton C, Prah P, Erens B, Sonnenberg P, Clifton S, Macdowall W, Lewis R, Field N, Datta J, Copas AJ, Phelps A, Wellings K, Johnson AM (2013). Changes in sexual attitudes and lifestyles in Britain through the life course and over time: findings from the national surveys of sexual attitudes and lifestyles (Natsal). Lancet.

[8] Sexually transmitted infections and screening for chlamydia in England, 2019. Public Health England.

[9] Simões D, Stengaard AR, Combs L, Raben D, EuroTEST COVID-19 impact assessment consortium of partners (2020). Impact of the COVID-19 pandemic on testing services for HIV, viral hepatitis and sexually transmitted infections in the WHO European Region, March to August 2020. Euro Surveill.

[10] Hodgson I (2020). European AIDS treatment group documents the impact of COVID on HIV services throughout Europe. NAM Aidsmap.

[11] Kelly MP, Barker M (2016). Why is changing health-related behaviour so difficult?. Public Health.

[12] (2015). Health promotion for sexual and reproductive health and HIV: strategic action plan, 2016 to 2019. Public Health England.

[13] Craig P, Dieppe P, Macintyre S, Michie S, Nazareth I, Petticrew M, Medical Research Council Guidance (2008). Developing and evaluating complex interventions: the new medical research council guidance. BMJ.

[14] Newby K, Crutzen R, Brown K, Bailey J, Saunders J, Szczepura A, Hunt J, Alston T, Sadiq ST, Das S (2019). An intervention to increase condom use among users of chlamydia self-sampling websites (wrapped): intervention mapping and think-aloud study. JMIR Form Res.

[15] Norton TR, Bogart LM, Cecil H, Pinkerton SD (2005). Primacy of affect over cognition in determining adult men's condom–use behavior: a review. J Appl Soc Psychol.

[16] Sheeran P, Abraham C, Orbell S (1999). Psychosocial correlates of heterosexual condom use: a meta-analysis. Psychol Bull.

[17] Noar SM, Carlyle K, Cole C (2006). Why communication is crucial: meta-analysis of the relationship between safer sexual communication and condom use. J Health Commun.

[18] Albarracín D, Kumkale GT, Johnson BT (2004). Influences of social power and normative support on condom use decisions: a research synthesis. AIDS Care.

[19] Eldridge SM, Chan CL, Campbell MJ, Bond CM, Hopewell S, Thabane L, Lancaster GA, PAFS consensus group (2016). CONSORT 2010 statement: extension to randomised pilot and feasibility trials. BMJ.

[20] Freetest.

[21] Woodhall SC, Sile B, Talebi A, Nardone A, Baraitser P (2012). Internet testing for chlamydia trachomatis in England, 2006 to 2010. BMC Public Health.

[22] Wrapped.

[23] Matomo.

[24] Teare MD, Dimairo M, Shephard N, Hayman A, Whitehead A, Walters SJ (2014). Sample size requirements to estimate key design parameters from external pilot randomised controlled trials: a simulation study. Trials.

[25] Free C, McCarthy O, French RS, Wellings K, Michie S, Roberts I, Devries K, Rathod S, Bailey J, Syred J, Edwards P, Hart G, Palmer M, Baraitser P (2016). Can text messages increase safer sex behaviours in young people? Intervention development and pilot randomised controlled trial. Health Technol Assess.

[26] University of Hertfordshire Research Archive.

[27] Gale NK, Heath G, Cameron E, Rashid S, Redwood S (2013). Using the framework method for the analysis of qualitative data in multi-disciplinary health research. BMC Med Res Methodol.

[28] Mitchell KR, Shimonovich M, Bosó Pérez R, Dema E, Clifton S, Riddell J, Copas AJ, Tanton C, Macdowall W, Bonell C, Sonnenberg P, Mercer CH, Field N (2023). Initial impacts of COVID-19 on sex life and relationship quality in steady relationships in Britain: findings from a large, quasi-representative survey (Natsal-COVID). J Sex Res.

[29] Dema E, Gibbs J, Clifton S, Copas AJ, Tanton C, Riddell J, Pérez RB, Reid D, Bonell C, Unemo M, Mercer CH, Mitchell KR, Sonnenberg P, Field N (2022). Initial impacts of the COVID-19 pandemic on sexual and reproductive health service use and unmet need in Britain: findings from a quasi-representative survey (Natsal-COVID). Lancet Public Health.

[30] Sonnenberg P, Menezes D, Freeman L, Maxwell KJ, Reid D, Clifton S, Tanton C, Copas A, Riddell J, Dema E, Bosó Pérez R, Gibbs J, Ridge MC, Macdowall W, Unemo M, Bonell C, Johnson AM, Mercer CH, Mitchell K, Field N (2022). Intimate physical contact between people from different households during the COVID-19 pandemic: a mixed-methods study from a large, quasi-representative survey (Natsal-COVID). BMJ Open.

