# Supplementary material for: An Intervention to Increase Condom Use Among Users of Sexually Transmitted Infection Self-sampling Websites (Wrapped): Protocol for a Randomized Controlled Feasibility Trial
Source: JMIR Res Protoc. 2023 May 11;12:e43645. doi: 10.2196/43645 (PMC10214115; doi:10.2196/43645)
Supplement: Multimedia Appendix 1 [file resprot_v12i1e43645_app1.doc]

Users of freetest.me click on link to study website placed on freetest.me ‘Thank You’ page (viewed after placing order for chlamydia self-test kit) (n= )

Excluded (n= )

♦  Not meeting inclusion criteria (n= )

♦ Declined to participate (n= )

♦ Did not complete consent (n=)

Users visit project page and assessed for eligibility (n=)

**Enrollment**

Users complete consent (n= )

Users complete baseline measures (n= /230)

♦ Did not complete baseline measures (n= )

Randomization (n= /230)

**Allocation**

**Assesment**

**Follow-up 3 Mths**

**Allocated to Usual Care* plus Wrapped^$^ (n= /115)**

♦ Participants do not access Wrapped via link (n= /115)

**Allocated to Usual Care* (n= 115)**

♦ Participants do not access STI info via link (n= /115)

♦ 2^0^ outcome measure (survey) not completed (n= )

♦  Withdrawal (n= /115)

        

♦ 1^0^ outcome measure (chlamydia sample) not provided (n= /115)

♦ 2^0^ measures (survey) not received (n= /115)

♦  Withdrawal (n= /115)

♦ 1^0^ outcome measure (chlamydia test result) not provided (n= /115)

♦ 1^0^ outcome measure (chlamydia test result) not provided (n= /115)

   

♦ 1^0^ outcome measure (chlamydia sample) not provided (n= /115)

♦ 2^0^ measures (survey) not received (n=/115)

♦  Withdrawal (n= /115)

**Follow-up 6 Mths**

♦ 2^0^ outcome measure (survey) not completed (n= )

♦  Withdrawal (n= /115)

**Follow-up 12 Mths**

**♦** **Loss to follow-up:** 1^0^ outcome measure

(chlamydia sample) not provided (n=/ 115)

♦ 2^0^ measures (survey) not received (n= /115)

♦  Withdrawal (n= /115)

**♦** **Loss to follow-up:** 1^0^ outcome measure

(chlamydia sample) not provided (n= /115)

♦ 2^0^ measures (survey) not received (n= /115)

♦  Withdrawal (n= /115)

     ♦ Assessed for objective i (n= /115)

♦ Assessed for objective i (n= /115) etc...

     ♦ Assessed for objective i (n= /115)

♦ Assessed for objective i (n= /115) etc...
