# Supplementary material for: An Intervention to Increase Condom Use Among Users of Sexually Transmitted Infection Self-sampling Websites (Wrapped): Protocol for a Randomized Controlled Feasibility Trial
Source: JMIR Res Protoc. 2023 May 11;12:e43645. doi: 10.2196/43645 (PMC10214115; doi:10.2196/43645)
Supplement: Multimedia Appendix 2 [file resprot_v12i1e43645_app2.doc]

**Multimedia appendix 2 – Schedule of voucher payments**

Amazon vouchers will be distributed by email to each participant upon completion of a data collection measure as follows:

- Month 0 (joining the study) £5
- Month 3 test kit £10
- Month 3 questionnaire £5
- Month 6 questionnaire £10
- Month 12 test kit £20
- Month 12 questionnaire £15
