# Supplementary material for: An Intervention to Increase Condom Use Among Users of Sexually Transmitted Infection Self-sampling Websites (Wrapped): Protocol for a Randomized Controlled Feasibility Trial
Source: JMIR Res Protoc. 2023 May 11;12:e43645. doi: 10.2196/43645 (PMC10214115; doi:10.2196/43645)
Supplement: Multimedia Appendix 3 [file resprot_v12i1e43645_app3.doc]

**Multimedia Appendix 3 – Advert schedule**

| **Advert number** | **Advert text** |
| --- | --- |
|  |  |
| 1 | Help us make a positive change to young people’s sexual health: make a difference by joining our study *and* get paid for your time! Click here to find out more. |
| 2 | Take part in a study to improve sexual health and earn up to £65 in Amazon vouchers, find out how you can make a difference today! Click here to find out more. |
| 3 | Your views are important: join our study to help improve sexual health for young people whilst being paid! Click here to find out more. |
| 4 | Make a difference to improve sexual health for young people - join our study and earn up to £65 in Amazon vouchers for your time! Click here to find out more. |
| 5 | Want to make a difference to young people’s sexual health? Take part in our study *and* get paid for your time! Click here to find out more. |
| 6 | We need your help! Take part in a study to improve young people’s sexual health and earn up to £65 in Amazon vouchers for your time. Click here to find out more. |
