# Supplementary material for: An Intervention to Increase Condom Use Among Users of Sexually Transmitted Infection Self-sampling Websites (Wrapped): Protocol for a Randomized Controlled Feasibility Trial
Source: JMIR Res Protoc. 2023 May 11;12:e43645. doi: 10.2196/43645 (PMC10214115; doi:10.2196/43645)
Supplement: Multimedia Appendix 4 [file resprot_v12i1e43645_app4.doc]

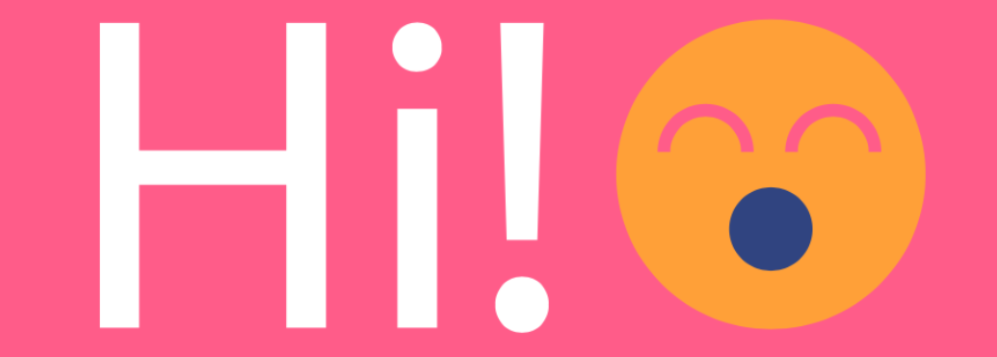


Participant Information

Thank you for your interest in our study!

Here is all the information you need to know about taking part. Please take time to read it carefully.

**Key Info:**

- We have developed a website to support young people to protect their sexual health
- This study aims to test whether young people will take part research to test whether this website works and complete all the activities involved
- The study lasts 12 months
- In this time you’ll be asked to:
  - complete four surveys (each takes about 10 mins)
  - take two additional chlamydia self-test kits (we will also ask you about the result of the one you’ve just ordered)
  - view one of two website (our new one and a control one)
- Each time you complete an activity, we’ll email you an Amazon e-voucher in return (see Detailed Info: ‘benefits of taking part’)
- All data will be kept confidential but there are some limits to that confidentiality (see Detailed Info: ‘limits to confidentiality’)
- All data will be handled in accordance with the General Data Protection Regulations (GDPR) and the Data Protection Act 2018 (DPA)
- There are some restrictions on who can take part – please complete the eligibility section below to see whether you meet these

**Detailed information**

**What is the full study title?**

Wrapped: A study to identify which strategies work best to recruit and retain participants in web-based sexual health research

**Can you tell me more about why you are doing this research?**

We have developed a website that aims to increase condom use amongst young people that use STI (sexually transmitted infections) self-testing websites (such as freetest.me). What we want to know is whether this website works. To find this out, we need to run a type of experiment called a Randomised Controlled Trial (RCT). Researchers sometimes find that they can’t attract enough people to take part in these types of studies, or that people drop out along the way, leading to poor quality data. Before we can go ahead with the RCT, we therefore need to demonstrate that we can attract young people to take part in this type of study and to keep them interested throughout. Would you be willing to help us with this?

**Do I have to take part?**

It is completely up to you whether or not you decide to take part in this study. What we do ask is that you carefully consider what’s involved first - this is described in the next section. Each of the activities that you’ll be asked to complete will require some commitment on your part, such as finding time to complete the online surveys and chlamydia test kits. These things are easy to do, they just take a little bit of time and dedication. We will reimburse you for this time (see benefits of taking part below). The success of this study depends on those involved completing all activities. *Therefore, if you feel like this might be too much for you to commit to over the next 12 months, we ask that you please don’t join the study*.

**Are there any age or other restrictions that may prevent me from participating?**

Yes, only people aged 16-24 years who have been invited to take part via an advert seen on freetest.me can take part in this study. Also, only those whose sexual activity includes penetrative sex (i.e. penis in vagina or anus) can take part. This is because sexually transmitted infections (STIs) are mostly passed in this way and this study is focused on reducing STIs.

**What will happen to me if I take part and how long will it take?**

The whole study takes 12 months to complete but the activities are *well spaced out*, and we will *reimburse you for your time* (see ‘benefits of taking part’ section below), so don’t be put off!

Here’s what we’ll ask you to do:

1. *Complete an initial online survey* (taking approx. 10 minutes) – this asks questions about you, your sexual orientation, your thoughts on condom use, your sexual behaviour (condom use), and any previous STI testing and results. We also will also ask you for some contact details at this point – this is needed so that we can for example, send you future surveys and post out chlamydia test kits.
2. *View a website* (time spent on this up to you) – you will be shown one of two websites (selected at random) which contain information on condom use. This website will be available to you throughout the entire study period (12 months) – we will send you a link to this in an email so that you can return as often as you like.
3. *Provide us with your freetest.me chlamydia test result* (takes 1 minute to complete) – this is the result of the chlamydia test that you have just requested via the freetest.me website. We will send you a text message in about 10 days asking you for this result. Don’t worry, this will be discreet (see the section: ‘What are the possible disadvantages, risks or side effects of taking part?’ for details on this). If the result is not known at this time, we will ask you again 10 days later. If you do not respond, we will send a further text message to try and obtain this information after which we will stop. If you test positive, we will also contact you by text to find out if you received treatment; as before, this will be discreet. If you don’t respond, we will send one further text to try and obtain this information. It is possible we may send you a few extra text messages based on your responses to help us understand your situation better.
4. *Take a second chlamydia test at 3 months* (takes approx. 5 minutes to complete)
5. *Complete first follow-up online survey also at 3 months* (takes approx. 10 minutes to complete)
6. *Complete second follow-up online survey at 6 months* (takes approx. 10 minutes to complete)
7. *Take a third chlamydia test at 12 months* (takes approx. 5 minutes to complete)
8. *Complete final follow-up online survey at 12 months* (takes approx. 10 minutes to complete)

*Info about the chlamydia tests at 3 and 12 months***:** we will send you a further freetest.me chlamydia self-test kit in the post (same discreet packaging). You will be asked to take a sample as before and return it to freetest.me in the freepost box provided. The result will be recorded by freetest.me in a secure service area which they have set up on their system especially for this project. We will access this service area daily (weekdays during working hours) and then text you the result the same day. If you test positive, we will confidentially and securely report this to your local NHS sexual health service who will contact you to arrange treatment. A few days later, we will contact you by text to find out if you received treatment. We will send up to 2 texts to try and obtain this information and then stop. It is possible we may send you a few extra text messages based on your responses to help us understand your situation better. All of our communication with you will be discreet (see the section: ‘What are the possible disadvantages, risks or side effects of taking part?’ for details on this). We may also make a friendly call to remind you to return the test or to provide us with information on the outcome of treatment.

*Info about the surveys at 3, 6 and 12 months*: a link to each will be sent via email at the appropriate timepoint. Three reminders (each 5 days apart) will be sent to those who do not complete the survey after receiving this email. Each survey will contain many of the same questions as the initial survey. That’s so that we can detect any changes that people experience in their condom use beliefs or behavior – so please don’t be put off by the repetitiveness, its deliberate! It is possible that we may make a friendly call to remind you to return a survey; we may use this opportunity to ask you a limited number of survey questions (just the most important stuff!).

*Optional extra*: All activities described above are voluntary. There is however an additional element of the study that some people will be invited to participate in *but only if they express an interest in this*. In other words, you can fully take part in the study without participating in this additional element. This additional element is a telephone or online ‘interview’ (lasting approx. 30 mins) which would take place at some time during the 12 month study period. The purpose of this would be to find out more about your views and experience of being involved in this research and of the website itself. As with the standard activities, we would reimburse you for your time (see benefits of taking part below). We will ask you if you are interested in taking part in this optional extra within the initial survey.

**What if I change my mind about taking part?**

We understand that life can sometimes get in the way of plans. If you can’t complete one of the activities, then it’s no big deal; we will simply carry on sending you invites to future activities so that you can continue to be involved. If for some reason however, you wish to withdraw altogether (and stop receiving the invitations to complete activities), then that’s no problem. Just email us at [wrapped@herts.ac.uk](mailto:wrapped@herts.ac.uk) to let us know – we won’t ask you for a reason for this, we’ll just respect your wishes. We will keep any data that you have provided up to that point and use it in our analyses unless you explicitly ask us not too; if you make this request and it is not possible to remove your data at that stage then we will inform you. Please be reassured that a decision to withdraw at any time, or a decision not to take part at all, will not affect the service that you receive from freetest.me or the NHS (should this be relevant). In the event of any significant change to the aim(s) or design of the study you will be informed and asked to renew your consent to participate in it.

**What are the possible disadvantages, risks or side effects of taking part?**

Taking part in this study requires you to complete surveys and chlamydia self-tests, respond to text messages, and to view a website about condom use. We therefore consider the risks of taking part to be low and in line with normal everyday activities. All communication with you will be discreet. For example, chlamydia test kits will be sent in plain packaging, email subject lines will not reveal what the study is about, and our text messages won’t refer to chlamydia testing or treatment specifically (they will say for example, ‘*You should now have received your test results. Click the link below to let us know the outcome*’ or ‘*You should now have been contacted about treatment following your test result. Can you tell us about any treatment received?*’). The website may lead you think about your own condom use and sexual health. Information will be provided on the website itself about how you can access condoms and support on issues relating to sexual health. An auto-generated email will also be sent to you on completion of each survey containing information on sources of further help and support should you need it.

Please note that as part of the study the only tests that will be carried out are for chlamydia. We are not testing for any other sexually transmitted infections (STI) which you may have had as part of your initial screen through freetest.me. If you want any additional testing, then you should visit your local sexual health service.

**What are the possible benefits of taking part?**

You will be given access to one of the two websites which you will be free to use as much as you like during the 12 month study. Also, you can feel rightly proud of yourself for contributing to important science - only with your input will it be possible for us to learn what will work to attract young people to a future RCT (Randomised Controlled Trial) and to keep them interested throughout. Finally, in recognition of the time and effort, you will receive Amazon vouchers for each completed activity as follows*:

- Month 0 (joining the study) £5
- Month 3 test kit £10
- Month 3 survey £5
- Month 6 survey £10
- Month 12 test kit £20
- Month 12 survey £15
- Optional extra (participation in interview): £20

*We will be running checks to satisfy ourselves that every participant is unique, eligible to participate, and takes reasonable time and care to answer survey questions. If we suspect that this is not the case, we reserve the right to prevent further participation and to withhold voucher payments.

**How will my taking part in this study be kept confidential?**

Your decision to take part and any data that you provide would be kept confidential. Details on how we will ensure this are provided in this section. Please note, there are circumstances under which we would break that confidentiality. Please see the ‘Are there any limits to confidentiality?’ section for further detail on this.

If you decide to take part, you will be asked to complete an online consent form on the next page. These forms will be completed and stored in ‘REDCap’ (you are viewing this information on REDCap now) a secure piece of software hosted on the University of Hertfordshire’s server, accessible only by the research team. We will not share these forms with anyone outside of the research team.

Whilst your name will be on this consent form, it will not be associated with any of your survey responses or test results held by us. Rather than using names, we will identify participants using a unique numerical ID. This will be assigned at the time of consent. This ID will be used throughout the process of the study to link survey responses, information about participants’ use of the websites, and chlamydia test results.

For the study, we need to collect info on your use of the website you are shown e.g. which pages you visit and how long you spend there. To reduce the burden on you, rather than ask you questions about this in our surveys, we will instead track your use using a specialist piece of software called Matomo. Matomo will record this along with your unique study ID and your IP address (restricted to 2 bytes e.g. 192.168.XXX.XXX).

Chlamydia test result data: As described above, we will be asking you to take two additional chlamydia self-tests; one 3 months into the study, and one at the end. Freetest.me will process these tests for us. The test kits will be similar to the one you have just requested. Freetest.me will record the results of these tests. We will access these test results by logging on to a secure service area of the freetest.me website. We will use your name and the kit code to identify you on this system, and then record this result on our REDCap database alongside your survey data. Any positive test results will be shared by us with the NHS sexual health service in your local area so that they can provide support/treatment. We (the University of Hertfordshire) and the NHS sexual health service in your local area have a legal Data Sharing Agreement in place to ensure that this data is treated with the upmost care and security.

**Are there any limits to confidentiality?**

Safeguarding means protecting a person’s right to live in safety, free from abuse and neglect. Everyone who takes part in this study will be provided with information on sources of help and support on issues relating to sexual wellbeing (via an automated email at end of each survey).

We do however have a particular responsibility to protect individuals under the age of 18 years. If you are under the age of 18 and reveal information during the study which indicates that you may be at risk of significant harm, our safeguarding procedure will be followed. By significant harm, we mean neglect, or physical, mental or emotional harm, or harm to your physical, mental or emotional well-being. There are questions in each survey which could alert us to this. We will highlight these to you so that you can choose whether or not to answer them. You may also reveal this to us if we ever have direct contact with you (e.g. though email, phone). In either case, our confidentiality agreement would end, and we would pass on the information that you have provided to the relevant safeguarding organisation(s) so that they can support you. You cannot refuse this referral process, but we would tell you that it was happening.

**Factors that might put others at risk**

If any circumstances, such as unlawful activity, become apparent during the study which we believe might have put someone else at risk, the University may refer the matter to the appropriate authorities and, under such circumstances, you will be withdrawn from the study.

**What will happen to the data collected within this study?**

All data will be handled in accordance with the General Data Protection Regulations (GDPR) and the Data Protection Act 2018 (DPA). Please click on [this link](https://www.herts.ac.uk/__data/assets/pdf_file/0004/317425/Wrapped-Privacy-Notice-FINAL-Dec-2020.pdf) to view/download our data privacy notice. Here are details of how each type of data will be used in this study:

Consent data- This data will be stored electronically on REDcap for the duration of data collection (12 months), after which it will be downloaded and stored within a folder on the University of Hertfordshire’s storage area for research data (R Drive) which is only accessible by the research team. At this point, the original data on REDCap will be deleted. The R drive is backed up daily, password protected, and behind the University’s Firewall. This consent data will be kept on the R drive for 6 years and then deleted.

Analytics data- This data will be stored in Matomo (located on a secure server) for the duration of data collection (12 months), after which it will be downloaded and stored on the R Drive alongside the survey data. At this point, the original data on Matomo will be deleted. At download, the IP data will be removed. At this point the analytics data will contain no identifying information i.e. it will be ‘anonymised’ and ready for analysis. Once analysis is complete, this analytics data will be moved from the R drive to UHRA for archiving (see next section for further details).

Survey data- This data will be stored electronically on REDCap for the duration of data collection (12 months), after which it will be downloaded, and stored on the R Drive. At this point, the original data on REDCap will be deleted. At download, the survey data will not contain any identifying information about you with the following two exceptions; 1) your postcode, and 2) a contact phone number (only for those who have indicated that they would like to take part in the additional interview (described above). Before storing the data, we will use your postcode to calculate an ‘Indices of Multiple Deprivation’ (IMD) score (you can find out more about what that score is [here](https://assets.publishing.service.gov.uk/government/uploads/system/uploads/attachment_data/file/833959/IoD2019_Infographic.pdf) if you’re interested), and then delete it. We will also remove your contact number (if provided) and store this in a separate file on the R drive along with your unique ID and name (we will use this later to make contact with those who would like to take part). At this point the survey data will contain no identifying information i.e. it will be ‘anonymised’ and ready for analysis. Once analysis is complete, the survey data will be moved from the R drive to UHRA for archiving (see next section for further details).

**Will the data be required for use in further studies?**

After we have finished our analysis, the anonymised survey and analytics data will be moved from the R Drive to the University of Hertfordshire’s Research Archive (UHRA). Here it will be ‘open access’, meaning that other researchers will be able to access it indefinitely in order to perform further analysis for the benefit of science.

**Who has reviewed this study?**

This study has been reviewed by:

- The University of Hertfordshire Health, Science, Engineering and Technology Ethics Committee with Delegated Authority (The UH protocol number is [insert])
- IRAS Project ID number [insert]

**Will I be contacted about the findings of the study?**

Periodically through the study, we will text you with a brief update on our progress. This will contain a link to a website so that you can read about this in more detail if wish to. Once we have completed our analysis (expected to be summer 2022), we will also produce a short written and video summary of what we have found. We will ask you if you want a copy of this in the final survey and if you do, we’ll send it to you via email.

**Who can I contact if I have any questions?**

Contact the study team by emailing [wrapped@herts.ac.uk](mailto:wrapped@herts.ac.uk) and we'll get back to you within 1-2 working days.

**Who should I contact if I have a complaint?**

Although we hope it is not the case, if you have any complaints or concerns about any aspect of the way you have been approached or treated during the course of this study, please contact the principal investigator, Dr Katie Newby, in the first instance by emailing [k.newby@herts.ac.uk](mailto:k.newby@herts.ac.uk).

If you are not satisfied with the response, please write to the University's Secretary and Registrar at the following address:

Secretary and Registrar

University of Hertfordshire

College Lane

Hatfield

Herts

AL10 9AB

Nearly there, please read the through the following statements…

**1]** I confirm that I have read the participant information and the [privacy notice](https://www.herts.ac.uk/__data/assets/pdf_file/0004/317425/Wrapped-Privacy-Notice-FINAL-Dec-2020.pdf)

**2]** I have been assured that it is my choice about whether to take part and understand that I may withdraw from the study at any time without disadvantage or having to give a reason.

**3]** I have been given information about the potential risks and benefits of taking part

**4]** I have been told how information relating to me (data obtained during the study, and data provided by me about myself) will be handled: how it will be kept secure, who will have access to it, and how it will or may be used, including that anonymised data will be deposited in a repository with open access (i.e. freely available).

**5]** I understand that website analytics will be used to track my use of the website I am shown as part of this study

**6]** I understand that the results of any chlamydia tests that I complete whilst taking part in this study (at months 3 and 12) will be shared by freetest.me with the research team

**7]** I understand that any positive chlamydia test results will be shared with my local NHS sexual health service

**8]** I understand the ways in which my taking part will be kept confidential but also that there are limits to that confidentiality

**9]** I understand that if there is any revelation of unlawful activity or any indication of non-medical circumstances that would or has put others at risk, the University may refer the matter to the appropriate authorities.

I agree to the above statements and consent to taking part in the study **Yes/ No**

First name

Surname

Signature

Date
