# Supplementary material for: An Intervention to Increase Condom Use Among Users of Sexually Transmitted Infection Self-sampling Websites (Wrapped): Protocol for a Randomized Controlled Feasibility Trial
Source: JMIR Res Protoc. 2023 May 11;12:e43645. doi: 10.2196/43645 (PMC10214115; doi:10.2196/43645)
Supplement: Multimedia Appendix 5 [file resprot_v12i1e43645_app5.doc]

**Required response*

| **Survey** | **Item** |
| --- | --- |
|  | **Your contact details** |
| Baseline | *Email address _________________________  *Mobile telephone number ____________________  *Postal address ________________________________________    *Which area do you live in?  🔾 East Sussex  🔾 Kingston-upon-Thames  🔾 Northamptonshire  🔾 Somerset  🔾 Warwickshire |
|  | **Optional extra?** |
| Baseline | Are you interested in taking part in the optional telephone or video call chat later on in this study?  🔾 Yes  🔾 No |
|  | **About you** |
| Baseline | *How old are you?  🔾 16  🔾 17  🔾 18  🔾 19  🔾 20  🔾 21  🔾 22  🔾 23  🔾 24 |
| Baseline | *How would you describe your ethnic background?  🔾Asian/Asian British  🔾Black or Black British  🔾Mixed/multiple ethnic groups  🔾White  🔾Other (please specify) ____________________ |
| Baseline | *How would you describe your gender?  🔾 Female  🔾 Male  🔾 Non-binary or gender fluid  🔾 Other (please specify) _____________________  *Is your gender identity the same as that assigned at birth?  🔾 Yes  🔾 No |
| Baseline | *How would you describe your sexual identity?  🔾Heterosexual  🔾Gay  🔾Lesbian  🔾Bisexual  🔾Asexual  🔾Other |
| Baseline | *How would you describe the financial situation of your family while growing up?  🔾 Very comfortable – I had everything I needed and more  🔾 Comfortable – money was never an issue  🔾 Fairly comfortable but it was necessary to keep an eye on money  🔾 Things were ok, but money was tight sometimes  🔾 Money seemed to be a problem a lot of the time  🔾 It was always a struggle to get the basics (food, clothes, heating) |
| Baseline  M3  M6  M12 | How would you describe your relationship status?  🔾 Single  🔾 In a casual sexual relationship with one person  🔾 In casual sexual relationships with two or more people  🔾 In a serious relationship with one person  🔾 In a serious relationship with more than one person  🔾 Combining serious and casual relationships  🔾 Other (please state) _____________________ |
|  | **Recent STI diagnosis** |
| Baseline | Have you been diagnosed with a sexually transmitted infection **in the last 3 months**?  🔾 Yes [*if selected, route to ‘a’ below*]  🔾 No  a) Which ones? Please select all that apply  🔾 No diagnosis in last 3 months  🔾 Genital warts  🔾 Herpes  🔾 Chlamydia [*if selected, route to ‘b’ below*]  🔾 Gonorrhoea  🔾 Public lice (crabs)  🔾 Trichomonas/TV  🔾 Non-specific urethritis (NSU)  🔾 Pelvic inflammatory disease  🔾 Syphilis  🔾 HIV  🔾 Hepatitis B or C  🔾 Yes, but cannot remember the name  🔾 Other (please specify) _____________________  b) How many times have you had a positive **chlamydia** result in the **last 3 months**?  🔾 Once [*if selected, route to ‘c’ below*]  🔾 Twice [*if selected, route to ‘d’ below*]  🔾 Three times [*if selected, route to ‘d’ below*]  🔾 Four or more times (say how many_______________) [*if selected, route to ‘d’ below*]  c. What happened with treatment?  🔾 I was prescribed antibiotics, and I took them all  🔾 I was prescribed antibiotics, but I didn’t take them all  🔾 I didn’t get a prescription for antibiotics  d. Please think about the *most recent* time you had Chlamydia. What happened with treatment?  🔾 I was prescribed antibiotics, and I took them all  🔾 I was prescribed antibiotics, but I didn’t take them all  🔾 I didn’t get a prescription for antibiotics |
| M3  M6  M12 | For 3-month survey:  About three months ago you requested a chlamydia home test kit from freetest.me. **Since that test**, have you been diagnosed with any sexually transmitted infections?  For 6-month survey:  About three months ago, we sent you a chlamydia home test kit in the post.  **Since that test**, have you been diagnosed with any sexually transmitted infections?  For 12-month survey:  About nine months ago, we sent you a chlamydia home test kit in the post.  **Since that test**, have you been diagnosed with any sexually transmitted infections?  🔾 Yes [*if selected, route to ‘a’ below*]  🔾 No  a) Which ones? Please select all that apply  🔾 No diagnoses since then  🔾 Genital warts  🔾 Herpes  🔾 Chlamydia [*if selected, route to ‘b’ below]*  🔾 Gonorrhoea  🔾 Public lice (crabs)  🔾 Trichomonas/TV  🔾 Non-specific urethritis (NSU)  🔾 Pelvic inflammatory disease  🔾 Syphilis  🔾 HIV  🔾 Hepatitis B or C  🔾 Yes, but cannot remember the name  🔾 Other (please specify) _____________________  b) In that period, how many times have you had a positive chlamydia result?  Please note, we only want to know about chlamydia, not any other STIs  🔾 Once [*if selected, route to ‘c’ below*]  🔾 Twice [*if selected, route to ‘d’ below*]  🔾 Three times [*if selected, route to ‘d’ below*]  🔾 Four or more times (say how many_______________) [*if selected, route to ‘d’ below*]  c. What happened with treatment for this infection?  🔾 I was prescribed antibiotics, and I took them all  🔾 I was prescribed antibiotics, but I didn’t take them all  🔾 I didn’t get a prescription for antibiotics  d. Please think about the *most recent* time you had Chlamydia. What happened with treatment?  🔾 I was prescribed antibiotics, and I took them all  🔾 I was prescribed antibiotics, but I didn’t take them all  🔾 I didn’t get a prescription for antibiotics |
|  | **Condom use – last few months** |
| Baseline  M3  M6  M12 | In the last few months, how often have you used a condom when having sex?  🔾 Every time  🔾 Almost every time  🔾 Sometimes  🔾 Occasionally  🔾 Never  In the last few months, how often did you not use a condom because your partner didn’t want to?  🔾 Every time  🔾 Almost every time  🔾 Sometimes  🔾 Occasionally  🔾 Never |
|  | **Condom use – next few months** |
| Baseline  M3  M6  M12 | Please rate your agreement with this statement  In the next few months I am going to use condoms every time I have sex  🔾 Strongly agree  🔾 Agree  🔾 Neither agree nor disagree  🔾 Disagree  🔾 Strongly disagree |
|  | **Condom practice** |
| Baseline  M3  M6  M12 | Have you ever practised putting on condoms in a class/ workshop (e.g. at school, college)?  🔾 Yes  🔾 No  Have you ever practised putting on condoms by yourself?  This could either be by putting them on yourself or on to a penis model, dildo etc  🔾 Yes [*if selected, route to ‘a’ below*]  🔾 No  a) How many times have you practised putting on condoms by yourself in the last few months?  🔾 Not in last few months  🔾 Once  🔾 2-3 times  🔾 4-6 times  🔾 7+ times  Have you ever experimented with different types of condoms to try and find one(s) that you like? E.g. in terms of fit, feel, texture, smell, pleasure etc  🔾 No I’ve never done this  🔾 Yes but I still haven’t found any I like  🔾 Yes and I have one(s) I like  🔾 Other (please give your response) __________________ |
| M3  M6  M12 | In the last 3 months, how many times have you practised putting on condoms by yourself in the last few months?  This could either be by putting them on yourself or on to a penis model, dildo etc  🔾 I haven’t done this  🔾 Once  🔾 2-3 times  🔾 4-6 times  🔾 7+ times  In the last 3 months, have you experimented with different types of condoms to try and find one(s) that you like?  E.g. in terms of fit, feel, texture, smell, pleasure etc  🔾 No I’ve never done this  🔾 Yes but I still haven’t found any I like  🔾 Yes and I have one(s) I like  🔾 Other (please give your response) __________________ |
| Baseline  M3  M6  M12 | In the last 3 months, have you experimented with different types of condoms to try and find ones that you like? E.g. in terms of fit, feel, texture, smell, pleasure etc  🔾 No  🔾 Yes but I still haven’t found any I like  🔾 Yes and I have one(s) I like  🔾 Other response you’d like to give__________________ |
| Baseline  M3  M6  M12 | When it comes to putting on condoms, how would you rate your current skill level?  By 'skill' we mean putting on a condom easily and effortlessly without making any mistakes  🔾 No skill at putting on condoms  🔾 A little skill  🔾 Somewhat skilled  🔾 Skilled  🔾 Very skilled |
|  | **How you feel about condoms** |
| Baseline  M3  M6  M12 | Please think about how you feel now and are likely to feel over the next few months  Using condoms every time I have sex is important to me.  🔾 Strongly agree  🔾 Agree  🔾 Neither agree nor disagree  🔾 Disagree  🔾 Strongly disagree  Sex with condoms can be pleasurable for me  🔾 Strongly agree  🔾 Agree  🔾 Neither agree nor disagree  🔾 Disagree  🔾 Strongly disagree  Sex with condoms can be pleasurable for my partner  🔾 Strongly agree  🔾 Agree  🔾 Neither agree nor disagree  🔾 Disagree  🔾 Strongly disagree  Sex with condoms can be enjoyable  🔾 Strongly agree  🔾 Agree  🔾 Neither agree nor disagree  🔾 Disagree  🔾 Strongly disagree  Using condoms will interrupt the flow of sex  🔾 Strongly agree  🔾 Agree  🔾 Neither agree nor disagree  🔾 Disagree  🔾 Strongly disagree  I (or my partner) don’t like the way condoms fit  🔾 Strongly agree  🔾 Agree  🔾 Neither agree nor disagree  🔾 Disagree  🔾 Strongly disagree  I don’t like the way that condoms feel  🔾 Strongly agree  🔾 Agree  🔾 Neither agree nor disagree  🔾 Disagree  🔾 Strongly disagree  Condoms are reliable protection against STIs  🔾 Strongly agree  🔾 Agree  🔾 Neither agree nor disagree  🔾 Disagree  🔾 Strongly disagree  Other people my age use condoms  🔾 Strongly agree  🔾 Agree  🔾 Neither agree nor disagree  🔾 Disagree  🔾 Strongly disagree |
|  | **Confidence with condoms** |
| Baseline  M3  M6  M12 | Please think about how you feel now and are likely to feel over the next few months  I am confident that:  I can correctly put a condom on myself or a partner  🔾 Strongly agree  🔾 Agree  🔾 Neither agree nor disagree  🔾 Disagree  🔾 Strongly disagree  I can discuss condom use with any partner I might have  🔾 Strongly agree  🔾 Agree  🔾 Neither agree nor disagree  🔾 Disagree  🔾 Strongly disagree  I can put a condom on myself or my partner without it ruining the mood  🔾 Strongly agree  🔾 Agree  🔾 Neither agree nor disagree  🔾 Disagree  🔾 Strongly disagree  I can get hold of more condoms when I need them  🔾 Strongly agree  🔾 Agree  🔾 Neither agree nor disagree  🔾 Disagree  🔾 Strongly disagree  I can say no to sex without condoms  🔾 Strongly agree  🔾 Agree  🔾 Neither agree nor disagree  🔾 Disagree  🔾 Strongly disagree |
|  | **Condom availability: last few months** |
| Baseline  M3  M6  M12 | Please think about how things have been over the *last few months*  I have made sure that I’ve always had plenty of condoms  🔾 Strongly agree  🔾 Agree  🔾 Neither agree nor disagree  🔾 Disagree  🔾 Strongly disagree  I have kept condoms in places where I’m most likely to have sex (e.g. bedroom)  🔾 Strongly agree  🔾 Agree  🔾 Neither agree nor disagree  🔾 Disagree  🔾 Strongly disagree  I have always had condoms on me when I’m out and about  🔾 Strongly agree  🔾 Agree  🔾 Neither agree nor disagree  🔾 Disagree  🔾 Strongly disagree  The cost of condoms has not been an issue for me  🔾 Strongly agree  🔾 Agree  🔾 Neither agree nor disagree  🔾 Disagree  🔾 Strongly disagree  I’ve had a way of easily getting hold of more condoms when I’m running low  🔾 Strongly agree  🔾 Agree  🔾 Neither agree nor disagree  🔾 Disagree  🔾 Strongly disagree |
|  | **Buying condoms and lube: last few months** |
| Baseline  M3  M6  M12 | Have you bought any condoms over the last 3 months?  🔾 Yes [*if selected, route to ‘a’ below*]  🔾 No  a) Approx how much would you say you’ve spent on condoms in the last 3 months? £_______________  Have you bought any lube over the last 3 months?  🔾 Yes [*if selected, route to ‘b’ below*]  🔾 No  b) Approx how much would you say you’ve spent on lube in the last 3 months? £_______________  Have you had any free condoms over the last 3 months?  🔾 Yes [*if selected, route to ‘c’ below*]  🔾 No  c) Approx how many free condoms would you say you’ve had over the last 3 months?:______________  Have you had any free sachets of lube over the last 3 months?  🔾 Yes [*if selected, route to ‘d’ below*]  🔾 No  d) How many free sachets of lube would you say you’ve had over the last 3 months? ________________ |
|  | **Condom availability: next few months** |
| Baseline  M3  M6  M12 | Please think about how you *feel now* and are likely to feel over the *next few months*  I know my preferred type of condom  🔾 Strongly agree  🔾 Agree  🔾 Neither agree nor disagree  🔾 Disagree  🔾 Strongly disagree  It is easy for me to keep condoms in places where I’m most likely to have sex  🔾 Strongly agree  🔾 Agree  🔾 Neither agree nor disagree  🔾 Disagree  🔾 Strongly disagree  It is easy for me to carry condoms when I’m out and about  🔾 Strongly agree  🔾 Agree  🔾 Neither agree nor disagree  🔾 Disagree  🔾 Strongly disagree  I have a way of letting a partner know that I want to use condoms that I’m happy with  🔾 Strongly agree  🔾 Agree  🔾 Neither agree nor disagree  🔾 Disagree  🔾 Strongly disagree  I am able to pick the right moment to let a partner know that I want to use condoms  🔾 Strongly agree  🔾 Agree  🔾 Neither agree nor disagree  🔾 Disagree  🔾 Strongly disagree  If I want to use condoms but my partner doesn’t, I know what to say  🔾 Strongly agree  🔾 Agree  🔾 Neither agree nor disagree  🔾 Disagree  🔾 Strongly disagree |
|  | **About your health and wellbeing** |
| Baseline  M3  M6  M12 | The questions over the next few pages may not seem all that relevant, but all of them are needed so please help us by answering them all if you can!  For each section, please select the answer that best describes your health today  MOBILITY  🔾 I have no problems in walking about  🔾 I have slight problems in walking about  🔾 I have moderate problems in walking about  🔾 I have severe problems in walking about  🔾 I am unable to walk about    SELF-CARE  🔾 I have no problems washing or dressing myself  🔾 I have slight problems washing or dressing myself  🔾 I have moderate problems washing or dressing myself  🔾 I have severe problems washing or dressing myself  🔾 I am unable to wash or dress myself    USUAL ACTIVITIES (e.g. work, study, housework, family or leisure activities)  🔾 I have no problems doing my usual activities  🔾 I have slight problems doing my usual activities  🔾 I have moderate problems doing my usual activities  🔾 I have severe problems doing my usual activities  🔾 I am unable to do my usual activities    PAIN / DISCOMFORT  🔾 I have no pain or discomfort  🔾 I have slight pain or discomfort  🔾 I have moderate pain or discomfort  🔾 I have severe pain or discomfort  🔾 I have extreme pain or discomfort    ANXIETY / DEPRESSION  🔾 I am not anxious or depressed  🔾 I am slightly anxious or depressed  🔾 I am moderately anxious or depressed  🔾 I am severely anxious or depressed  🔾 I am extremely anxious or depressed |
|  | Please use the slider to tell us how good or bad your health is today  0 = worst health you can imagine  100 = best health you can imagine |
|  | 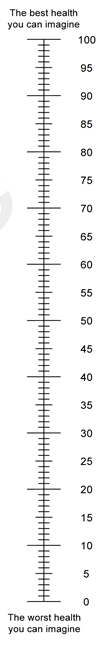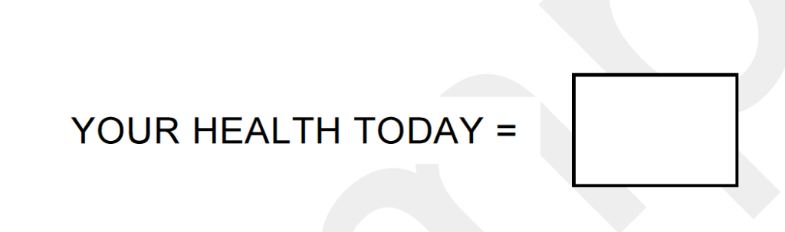 |
|  | SF-12v2® Health Survey © 1992, 2002,2013Medical Outcomes Trust and Quality Metric Incorporated.  All rights reserved.  SF-12® is a registered trademark of Medical Outcomes Trust. (SF-12v2®Health Survey Standard, United Kingdom (English)  Your Health and Well-Being  This survey asks for your views about your health. This information will help keep track of how you feel and how well you are able to do your usual activities. Thank you for completing this survey!  For each of the following questions, please select the one response that best describes your answer. |
|  | In general, would you say your health is:  🔾 Excellent  🔾 Very good  🔾 Good  🔾 Fair  🔾 Poor |
|  | The following questions are about activities you might do during a typical day. Does your health now limit you in these activities? If so, how much?   \|  \| YES, limited a lot \| YES, limited a little \| NO, not limited at all \| \| --- \| --- \| --- \| --- \| \| Does your health now limit you in moderate activities, such as moving a table, pushing a vacuum cleaner, bowling, or playing golf? If so, how much? \| 🔾 \| 🔾 \| 🔾 \| \| Does your health now limit you in climbing several flights of stairs? If so, how much? \| 🔾 \| 🔾 \| 🔾 \| |
|  | During the past 4 weeks, have you had any of the following problems with your work or other regular daily activities as a result of your physical health?     \|  \| All of the time \| Most of the time \| Some of the time \| A little of the time \| None of the time \| \| --- \| --- \| --- \| --- \| --- \| --- \| \| During thepast4weeks, how much of the time have you accomplished less than you would have liked as a result of your physical health? \| 🔾 \| 🔾 \| 🔾 \| 🔾 \| 🔾 \| \| During the past 4 weeks, how much of the time were you limited in the kind of work or other activities you do as a result of your physical health? \| 🔾 \| 🔾 \| 🔾 \| 🔾 \| 🔾 \|   During the past 4 weeks, have you had any of the following problems with your work or other regular daily activities as a result of any emotional problems (such as feeling depressed or anxious)?   \|  \| All of the time \| Most of the time \| Some of the time \| A little of the time \| None of the time \| \| --- \| --- \| --- \| --- \| --- \| --- \| \| During thepast4weeks, how much of the time have you accomplished less than you would have liked as a result of any emotional problems (such as feeling depressed or anxious)? \| 🔾 \| 🔾 \| 🔾 \| 🔾 \| 🔾 \| \| During the past 4 weeks, how much of the time did you do work or other activities less carefully than usual as a result of any emotional problems (such as feeling depressed or anxious)? \| 🔾 \| 🔾 \| 🔾 \| 🔾 \| 🔾 \|   During the past 4 weeks, how much did pain interfere with your normal work (including work outside the home and housework)?  🔾1 Not at all  🔾2 A little bit  🔾3 Moderately  🔾4 Quite a bit  🔾5 Extremely  These questions are about how you have been feeling during the past 4 weeks. For each question, please give the one answer that comes closest to the way you have been feeling.   \|  \| All of the time \| Most of the time \| A good bit of the time \| Some of the time \| A little bit of the time \| None of the time \| \| --- \| --- \| --- \| --- \| --- \| --- \| --- \| \| How much of the time during the past 4 weeks have you felt calm and peaceful? \| 🔾 \| 🔾 \| 🔾 \| 🔾 \| 🔾 \| 🔾 \| \| How much of the time during the past 4 weeks did you have a lot of energy? \| 🔾 \| 🔾 \| 🔾 \| 🔾 \| 🔾 \| 🔾 \| \| How much of the time during the past 4 weeks have you felt downhearted and low? \| 🔾 \| 🔾 \| 🔾 \| 🔾 \| 🔾 \| 🔾 \|   During the past 4 weeks, how much of the time has your physical health or emotional problems interfered with your social activities (like visiting friends, relatives, etc.)?  🔾1 All of the time  🔾2 Most of the time  🔾3 Some of the time  🔾4 A little of the time  🔾5 None of the time |
|  | **Your use of online STI self-testing services** |
| Baseline  M3  M6  M12 | *Wording for baseline:*  Apart from the home test kit you’ve just ordered from freetest.me, have you  ordered any other free home STI test kits from a STI testing website in the last 3 months?  *Wording for 3M, 6M and 12M:*  In the last 3 months, have you ordered any free home STI test kits from a free STI testing website?  🔾 Yes [*if selected, route to ‘b’ below*]  🔾 No  b) Select all of the free STI test kits you've ordered in the last 3 months:  🔾 A kit that tests for chlamydia only  🔾 A kit that tests for gonorrhoea only  🔾 A kit that tests for both chlamydia and gonorrhoea  🔾 A kit which tests for HIV only  🔾 A full screening kit (chlamydia, gonorrhoea HIV and syphilis)  🔾 Other  [*For each of the above selected the following is displayed*]  Total number ordered _________  Did you return them all?  🔾 Yes  🔾 No [*if selected, route to ‘c’ below*]  c) Total number returned _________  Have you paid for any STI test kits online in the last 3 months?  Total amount paid for STI testing kit(s) online, in the last three months (your best guess will do) £______________ |
|  | **Your use of NHS Healthcare services** |
| Baseline  M3  M6  M12 | In the last 3 months, have you had any contact with an NHS service in connection with your sexual health (e.g. following a positive STI test or for a check-up)?  🔾 Yes (display a)  🔾 No  a) Select all of the NHS services that you’ve had contact with in the last 3 months  🔾 Sexual health clinic (GUM clinic)  🔾 GP appointment  🔾 GP nurse appointment  🔾 NHS walk-in centre  🔾 NHS 111 / NHS direct call (phone service)  🔾 GP out of hours service  🔾 Pharmacy (general appointment)  🔾 A&E Department  🔾 Outpatient visit (e.g. to see gynaecologist, urologist)  🔾 Other (please write in)  [*For each of the above selected, the following is displayed*]  Total number of face-to-face contacts __________  Total number of telephone or video contacts __________ |
|  | **Services provided by your school / college / University** |
| Baseline  M3  M6  M12 | Are you currently attending school, college or university?  This could be full-time or part-time  🔾 Yes [*if selected, route to a below]*  🔾 No  a) In the last 3 months, have you accessed any services at your school, college or university in relation to your sexual health?  🔾 Yes [*if selected, route to b below]*  🔾 No  b) Please select all of the services you have accessed  🔾 School / college / university nurse  🔾 School / college / university welfare / support worker  🔾 Psychologist  🔾 Counsellor  🔾 Extra classes  🔾 Other  [*For each of the above selected, the following is displayed*]  Total number of face-to-face contacts __________  Total number of telephone or video contacts __________ |
|  | **Your use of treatment, medicines or services** |
| Baseline  M3  M6  M12 | Which of the following have you received in the last 3 months (tick all that apply)?  🔾 Contraceptive pill [*if selected, route to a below]*  🔾 Anti-HIV prophylaxis/ Post-exposure prophylaxis (PEP) [*if selected, route to b below]*  🔾 Emergency contraception (morning after pill) [*if selected, route to c below]*  🔾 Counselling [*if selected, route to d below]*  🔾 Other [*if selected, route to e & f below]*  a) For any contraceptive pills received in the last 3 months, was this NHS prescribed (free) or did you pay for them?  🔾 I paid for it [*if selected, route to a i below]*  🔾 It was prescribed/given to me for free by the NHS [*if selected, route to a ii below]*  🔾 Both of the above [*if selected, route to a i & ii below]*  a i) Total amount paid for the contraceptive pill in the last 3 month (your best guess will do)  a ii) How many times were you prescribed/given the contraceptive pill for free by the NHS in the last 3 months?  b) For any PEP received in the last 3 months, was this NHS prescribed (free) or did you pay for it?  🔾 I paid for it [*if selected, route to b i below]*  🔾 It was prescribed/given to me for free by the NHS [*if selected, route to b ii below]*  🔾 Both of the above [*if selected, route to b i & ii below]*  b i) Total amount paid for PEP in the last 3 month (your best guess will do)  b ii) How many times were you prescribed/given PEP for free by the NHS in the last 3 months?  c) For any emergency contraception received in the last 3 months, was this NHS prescribed (free) or did you pay for it?  🔾 I paid for it [*if selected, route to c i below]*  🔾 It was prescribed/given to me for free by the NHS [*if selected, route to c ii below]*  🔾 Both of the above [*if selected, route to c i & ii below]*  c i) Total amount paid for emergency contraception in the last 3 month (your best guess will do)  c ii) How many times were you prescribed/given emergency contraception for free by the NHS in the last 3 months?  d) For any counselling received in the last 3 months, was this NHS prescribed (free) or did you pay for it?  🔾 I paid for it [*if selected, route to d i below]*  🔾 It was prescribed/given to me for free by the NHS [*if selected, route to d ii below]*  🔾 Both of the above [*if selected, route to d i & ii below]*  d i) Total amount paid for counselling in the last 3 month (your best guess will do)  d ii) How many times were you prescribed/given counselling for free by the NHS in the last 3 months?  e) Please tell us about the other treatment you received  f) For this other treatment received in the last 3 months, was this NHS prescribed (free) or did you pay for it?  🔾 I paid for it [*if selected, route to f i below]*  🔾 It was prescribed/given to me for free by the NHS [*if selected, route to f ii below]*  🔾 Both of the above [*if selected, route to f i & ii below]*  f i) Total amount paid for this other treatment in the last 3 month (your best guess will do)  f ii) How many times were you prescribed/given this other treatment for free by the NHS in the last 3 months? |
|  | Sexual wellbeing – abuse, assault and coercion |
| Baseline  M3  M6  M12 | Important, please read   - The next four questions ask about whether you have experienced unwanted sex and other types of illegal and unacceptable behaviour by a partner - If you are *under 18*, we have a responsibility to protect you. This means that if you tell us that you have been a victim, we will pass your contact details on to relevant organisation(s) in your local area who will try to get in touch so that they can work with you to find out what help (if any) is needed - You can choose not to answer these questions. Everyone, including those who do not answer, will receive an email on completion of this survey containing links to organisations that can help   In the last 3 months, how often have you had sex in exchange for something (e.g. drugs, alcohol, money, food, a place to sleep, gifts)?  🔾 never 🔾 1-2 times 🔾 3-6 times 🔾 7 or more times  In the last 3 months, how often have you had sexual contact that you did not want?  🔾 never 🔾 1-2 times 🔾 3-6 times 🔾 7 or more times  In the last 3 months, how often have you experienced verbal aggression or abuse from a sexual partner?  🔾 never 🔾 1-2 times 🔾 3-6 times 🔾 7 or more times  In the last 3 months, how often have you experienced physical aggression or assault by a sexual partner?  🔾 never 🔾 1-2 times 🔾 3-6 times 🔾 7 or more times |
|  | **Experience of website** |
| M3 | After completing the first survey, we emailed you a link to a website about condom use called ‘Wrapped’. Did you visit this?  [screenshot of website shown here]  🔾Yes [*if selected, route to a & b below]*  🔾No  a) How useful did you find this website?  🔾 Not at all useful  🔾 Slightly useful  🔾 Moderately useful  🔾 Very useful  🔾 Extremely useful  b) How would you rate the design/usability of this website?  🔾 Very poor  🔾 Poor  🔾 Average  🔾 Good  🔾 Very good |
|  |  |
| M3  M6  M12 | Wording for M3:  Since completing the first survey, has taking part in this study caused any problems for you at all? Please select all that apply and add anything you have experienced that isn't listed  Wording for M6 & M12:  Since the last survey you completed for us, has taking part in this study caused any problems for you at all? Please select all that apply and add anything you have experienced that isn't listed  🔾It has led to my parent or another person finding out I was having sex when I didn’t want them to know  🔾It has led to my parent or another person finding out I was getting tested for an STI when I didn’t want them to know  🔾It has led me to begin using, or increased my use of, pornography  🔾Other problem (please tell us about this) __________________________ |
|  | **Other websites about condom use** |
| M12 – control participants | Below are some screenshots from a website which provides information and support on condom use. Do you recognise any of these images?  [*display selection of x4 images from the Wrapped intervention website: home page, condom sample pack, condom carrier, condom demo*]  🔾Yes, I recognise some or all of them  🔾No, I don’t recognise this at all |
|  | **Your use of the items from the Wrapped website** |
| M12 – intervention participants | Did you receive any of the following items via the Wrapped website^$^?  Please select all that apply [images of each displayed]  ^$^Please note: different people were given access to different items from the Wrapped website depending on their needs. You therefore may not have had the chance to order all of these items  🔾Sample box of condoms and lube [*if selected, route to a-d below]*  🔾Monthly delivery of condoms and lube [*if selected, route to e below]*  🔾Condom carrier [*if selected, route to h below]*   1. Did you try any of the condoms?   🔾Yes  🔾No   1. Did you try any of the lube?   🔾Yes  🔾No   1. Did you look at the leaflet that came inside the box?   🔾Yes  🔾No   1. Have you used the box for storing condoms?   🔾Yes  🔾No   1. How many times did you place an order?   🔾Once (if selected, display f)  🔾More than once (if selected, display g)   1. How much of the condoms/lube have you used?   🔾I used all of it  🔾I used some of it  🔾I used most of it  🔾I haven’t used any of it   1. How much of the condoms/lube in your **most recent** order have you used?   🔾I used all of it  🔾I used some of it  🔾I used most of it  🔾I haven’t used any of it   1. Have you used the carrier?   🔾Yes, I use it to carry condoms (*if selected, display i*)  🔾Yes, but I don’t use it to carry condoms (*if selected, display i)*  🔾No, I don’t use it (*if selected, display j)*   1. Is the carrier attached to your keys/bag?   🔾Yes  🔾No   1. Why is this? (select all that apply)   🔾I didn’t like the colour  🔾I didn’t like the design  🔾Not discrete enough  Other reason (please tell us this) ___________________ |
|  | **Just a couple of last things from us…** |
| M12 | If you have any comments (good or bad) about participating in this study or about any of the surveys, we are keen to hear them. Please use the space below to provide any feedback that you would like to give |
| M12 | Once we have completed our analysis (expected to be summer 2022), we will produce a short written and video summary of what we have found. Would you like to be sent a copy of this via email?  🔾Yes  🔾No |
