# Supplementary material for: An Intervention to Increase Condom Use Among Users of Sexually Transmitted Infection Self-sampling Websites (Wrapped): Protocol for a Randomized Controlled Feasibility Trial
Source: JMIR Res Protoc. 2023 May 11;12:e43645. doi: 10.2196/43645 (PMC10214115; doi:10.2196/43645)
Supplement: Multimedia Appendix 6 [file resprot_v12i1e43645_app6.pptx]

## Slide 1
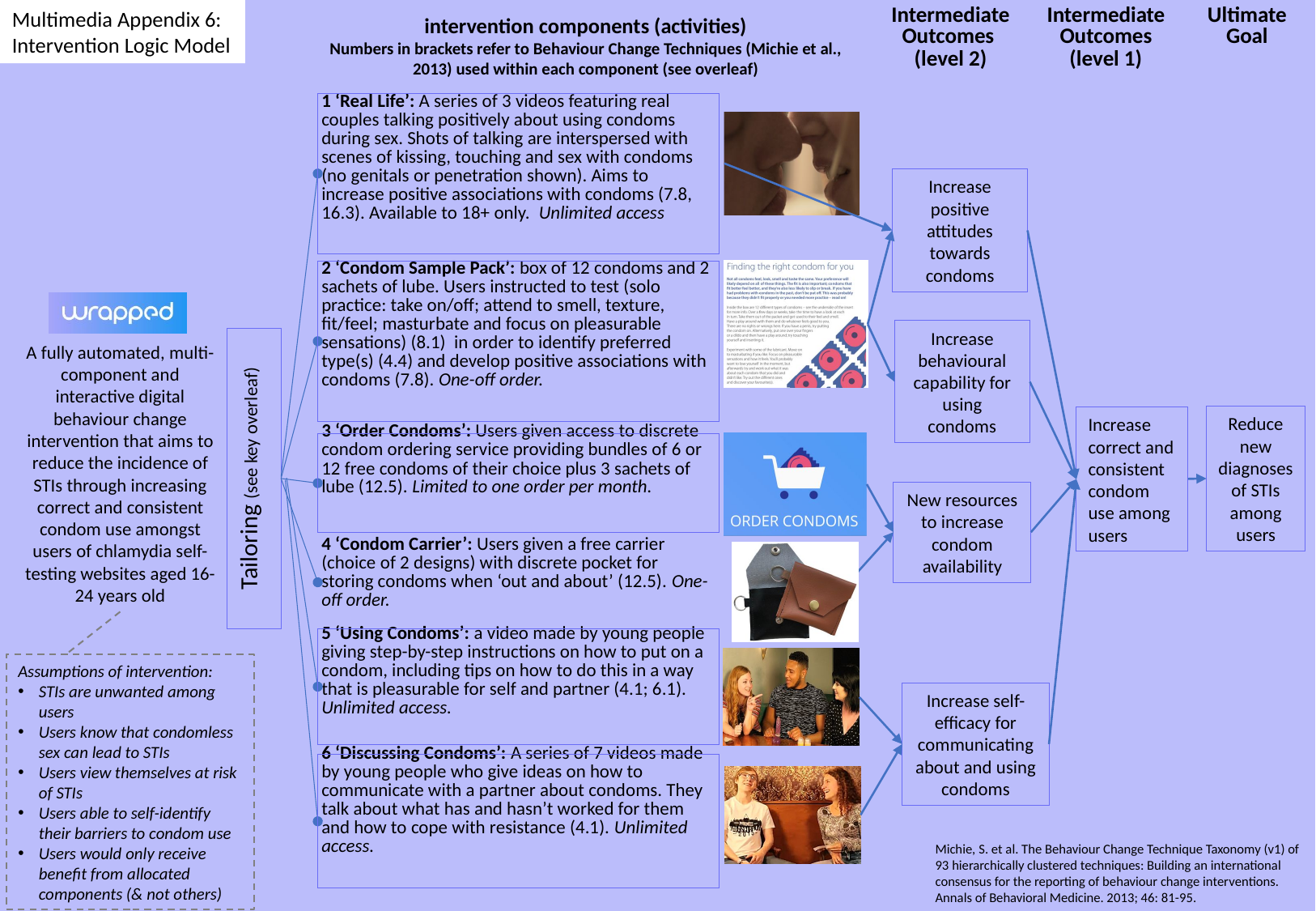

Multimedia Appendix 6: Intervention Logic Model
| | | | Intermediate Outcomes (level 2) | Intermediate Outcomes (level 1) | Ultimate Goal |
| --- | --- | --- | --- | --- | --- |
| | 1 ‘Real Life’: A series of 3 videos featuring real couples talking positively about using condoms during sex. Shots of talking are interspersed with scenes of kissing, touching and sex with condoms (no genitals or penetration shown). Aims to increase positive associations with condoms (7.8, 16.3). Available to 18+ only. Unlimited access | | | | |
| | ‘Condom Sample Pack’: box of 12 condoms and 3 sachets lube. Users instructed to test (solo practice: take on/off; attend to smell, texture, fit/feel; masturbate and focus on pleasurable sensations) (8.1) in order to identify preferred type(s) (4.4) and develop positive associations with condoms (7.8) . One-off order. | | | | |
| | 2 ‘Condom Sample Pack’: box of 12 condoms and 2 sachets of lube. Users instructed to test (solo practice: take on/off; attend to smell, texture, fit/feel; masturbate and focus on pleasurable sensations) (8.1) in order to identify preferred type(s) (4.4) and develop positive associations with condoms (7.8). One-off order. | | | | |
| | 3 ‘Order Condoms’: Users given access to discrete condom ordering service providing bundles of 6 or 12 free condoms of their choice plus 3 sachets of lube (12.5). Limited to one order per month. | | | | |
| | 4 ‘Condom Carrier’: Users given a free carrier (choice of 2 designs) with discrete pocket for storing condoms when ‘out and about’ (12.5). One-off order. | | | | |
| | 5 ‘Using Condoms’: a video made by young people giving step-by-step instructions on how to put on a condom, including tips on how to do this in a way that is pleasurable for self and partner (4.1; 6.1). Unlimited access. | | | | |
| | 6 ‘Discussing Condoms’: A series of 7 videos made by young people who give ideas on how to communicate with a partner about condoms. They talk about what has and hasn’t worked for them and how to cope with resistance (4.1). Unlimited access. | | | | |
intervention components (activities)
Numbers in brackets refer to Behaviour Change Techniques (Michie et al., 2013) used within each component (see overleaf)
Increase positive attitudes towards condoms
Increase behavioural capability for using condoms
Tailoring (see key overleaf)
A fully automated, multi-component and interactive digital behaviour change intervention that aims to reduce the incidence of STIs through increasing correct and consistent condom use amongst users of chlamydia self-testing websites aged 16-24 years old
Reduce new diagnoses of STIs among users
Increase correct and consistent condom use among users
New resources to increase condom availability
Assumptions of intervention:
STIs are unwanted among users
Users know that condomless sex can lead to STIs
Users view themselves at risk of STIs
Users able to self-identify their barriers to condom use
Users would only receive benefit from allocated components (& not others)
Increase self-efficacy for communicating about and using condoms
Michie, S. et al. The Behaviour Change Technique Taxonomy (v1) of 93 hierarchically clustered techniques: Building an international consensus for the reporting of behaviour change interventions. Annals of Behavioral Medicine. 2013; 46: 81-95.

## Slide 2
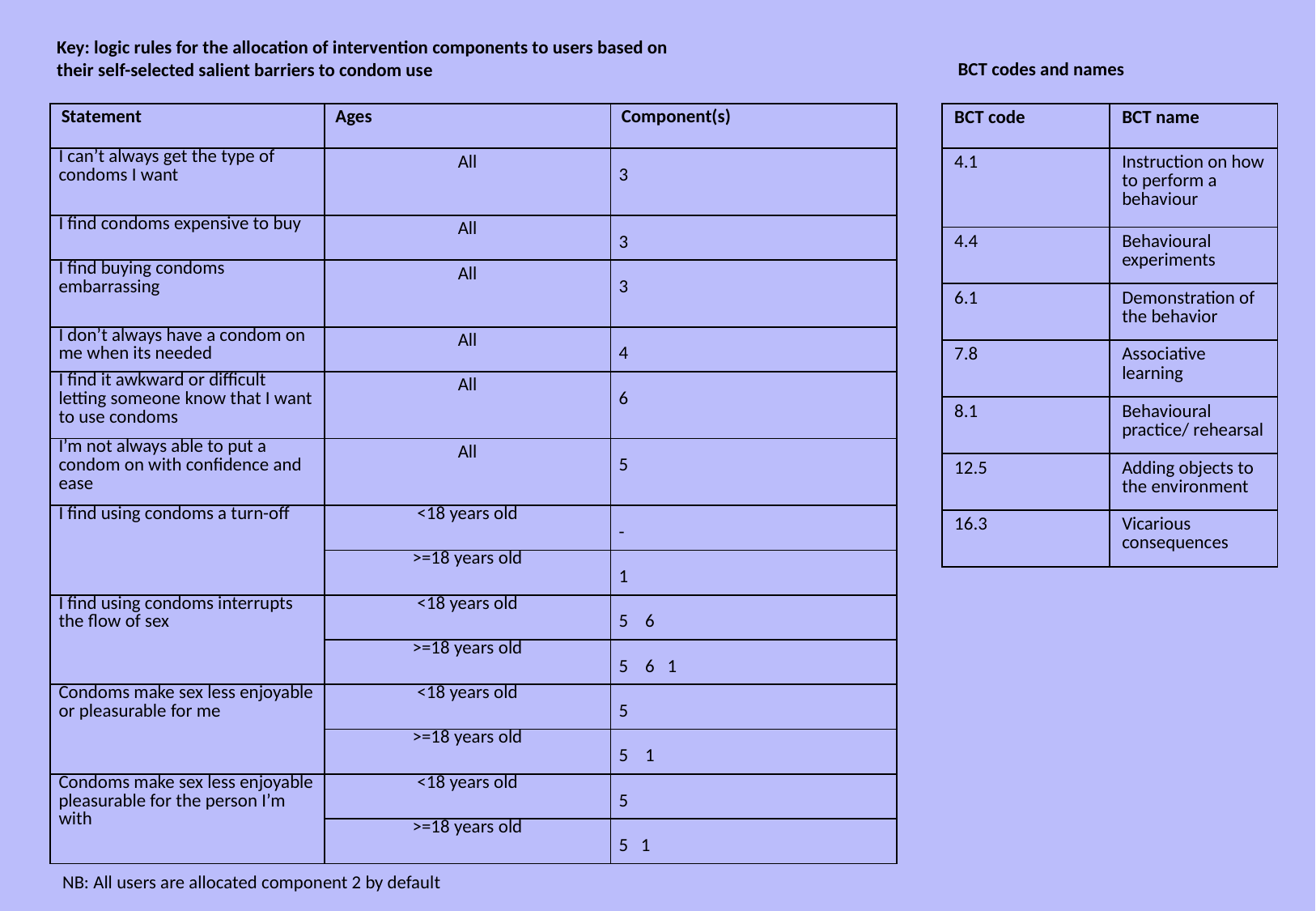

Key: logic rules for the allocation of intervention components to users based on their self-selected salient barriers to condom use
BCT codes and names
| Statement | Ages | Component(s) |
| --- | --- | --- |
| I can’t always get the type of condoms I want | All | 3 |
| I find condoms expensive to buy | All | 3 |
| I find buying condoms embarrassing | All | 3 |
| I don’t always have a condom on me when its needed | All | 4 |
| I find it awkward or difficult letting someone know that I want to use condoms | All | 6 |
| I’m not always able to put a condom on with confidence and ease | All | 5 |
| I find using condoms a turn-off | <18 years old | - |
| | >=18 years old | 1 |
| I find using condoms interrupts the flow of sex | <18 years old | 5 6 |
| | >=18 years old | 5 6 1 |
| Condoms make sex less enjoyable or pleasurable for me | <18 years old | 5 |
| | >=18 years old | 5 1 |
| Condoms make sex less enjoyable pleasurable for the person I’m with | <18 years old | 5 |
| | >=18 years old | 5 1 |
| BCT code | BCT name |
| --- | --- |
| 4.1 | Instruction on how to perform a behaviour |
| 4.4 | Behavioural experiments |
| 6.1 | Demonstration of the behavior |
| 7.8 | Associative learning |
| 8.1 | Behavioural practice/ rehearsal |
| 12.5 | Adding objects to the environment |
| 16.3 | Vicarious consequences |
NB: All users are allocated component 2 by default
