# Supplementary material for: An Intervention to Increase Condom Use Among Users of Sexually Transmitted Infection Self-sampling Websites (Wrapped): Protocol for a Randomized Controlled Feasibility Trial
Source: JMIR Res Protoc. 2023 May 11;12:e43645. doi: 10.2196/43645 (PMC10214115; doi:10.2196/43645)
Supplement: Multimedia Appendix 7 [file resprot_v12i1e43645_app7.doc]

**Multimedia Appendix 7 – analytics data measurements**

For each participant in the intervention condition we will record:

- Which components were allocated
- Which component pages were accessed (how many times and how long each visit was)
- Whether order placed for condom sample pack (event)
- Whether order placed for condom carrier (if allocated; event)
- Whether (first) order placed for condom delivery (if allocated; event); and whether any subsequent orders placed (frequency)
- Whether a condom review left
- Whether ‘using condoms’ video watched in full (or time watched for)
- How many of ‘discussing condoms’ videos watched (in full; or in part how long for)
- How many of ‘real life’ videos watched (in full; or in part how long for)
